# Allele-aware chromosome-level genome assembly and efficient transgene-free genome editing for the autotetraploid cultivated alfalfa

**DOI:** 10.1038/s41467-020-16338-x

**Published:** 2020-05-19

**Authors:** Haitao Chen, Yan Zeng, Yongzhi Yang, Lingli Huang, Bolin Tang, He Zhang, Fei Hao, Wei Liu, Youhan Li, Yanbin Liu, Xiaoshuang Zhang, Ru Zhang, Yesheng Zhang, Yongxin Li, Kun Wang, Hua He, Zhongkai Wang, Guangyi Fan, Hui Yang, Aike Bao, Zhanhuan Shang, Jianghua Chen, Wen Wang, Qiang Qiu

**Affiliations:** 1 State Key Laboratory of Genetic Resources and Evolution, Kunming Institute of Zoology, Chinese Academy of Sciences, 650223 Kunming, China; 2 Guangdong Sanjie Forage Biotechnology Co., Ltd., 510630 Guangzhou, China; 3 Sanjie Institute of Forage, 712100 Yangling, China; 4 Kunming College of Life Science, University of Chinese Academy of Sciences, 650204 Kunming, China; 5 University of Chinese Academy of Sciences, 100049 Beijing, China; 6 State Key Laboratory of Grassland Agro-Ecosystem, Lanzhou University, 730000 Lanzhou, China; 7 School of Ecology and Environment, Northwestern Polytechnical University, 710072 Xi’an, China; 8 BGI-Qingdao, 266555 Qingdao, China; 9 Center of Special Environmental Biomechanics & Biomedical Engineering, School of Life Sciences, Northwestern Polytechnical University, 710072 Xi’an, China; 10 CAS Key Laboratory of Tropical Plant Resources and Sustainable Use, CAS Center for Excellence in Molecular Plant Sciences, Xishuangbanna Tropical Botanical Garden, 650223 Kunming, China

**Keywords:** Agricultural genetics, Genomics, Molecular engineering in plants

## Abstract

Artificially improving traits of cultivated alfalfa (*Medicago sativa* L.), one of the most important forage crops, is challenging due to the lack of a reference genome and an efficient genome editing protocol, which mainly result from its autotetraploidy and self-incompatibility. Here, we generate an allele-aware chromosome-level genome assembly for the cultivated alfalfa consisting of 32 allelic chromosomes by integrating high-fidelity single-molecule sequencing and Hi-C data. We further establish an efficient CRISPR/Cas9-based genome editing protocol on the basis of this genome assembly and precisely introduce tetra-allelic mutations into null mutants that display obvious phenotype changes. The mutated alleles and phenotypes of null mutants can be stably inherited in generations in a transgene-free manner by cross pollination, which may help in bypassing the debate about transgenic plants. The presented genome and CRISPR/Cas9-based transgene-free genome editing protocol provide key foundations for accelerating research and molecular breeding of this important forage crop.

## Abstract (web summary)

Alfalfa is an important forage crop, but genetic improvement is challenging due to the lack of a reference genome and an efficient genome editing protocol. Here, the authors report the chromosome-level assembly of the autotetraploid genome and a CRISPR/Cas9-based transgene-free genome editing protocol.

## Introduction

Cultivated alfalfa (*Medicago sativa* L.) is a perennial herbaceous legume that has been cultivated since at least ancient Greek and Roman times^1^. It is one of the world’s most important forage species, due to its high nutritional quality, yields, and adaptability^1^. As a major forage protein source for livestock, alfalfa is cultivated over 80 countries with coverage exceeding 30 million hectares^1,2^. It is the third most valuable (7.8–10.8 billion dollars) and the fourth most widely grown (8.7 million hectares) field crop in the USA, after corn, soybean, and wheat^3^. Rapid increases in livestock production have also greatly increased demands for alfalfa forage in developing countries such as China in the last 50 years^4^. In addition to its high value as fodder, alfalfa cultivation is important for improving soil quality in appropriate areas^5,6^. Therefore, alfalfa has the potential to improve global food security as well as being a commercially valuable crop in its own right^7^.

However, cultivated alfalfa is a self-incompatibly cross-pollinated autotetraploid (2*n* = 4× = 32) plant with tetrasomic inheritance in which bivalent pairing is random and not preferential^8,9^, giving rise to a very complex genome that hinders efforts to decipher it genome and improve its traits. Previous exploration of genetic and genomic resources of alfalfa mostly relies on its close relative, the diploid *M. truncatula* (2*n* = 2×=16 = 860 Mb) which has been sequenced^10–12^. However, this has obvious limitations because they are different species and have different genomes. The assembly of autopolyploid genomes is severely hindered by the high similarity of their subgenomes and large genome size^13^. So far, only five plant autopolyploid genomes have been reported. Of these five, only the sugarcane *Saccharum spontaneum* genome was assembled de novo to the chromosome level using Hi-C data^14^, and the sweet potato (*Ipomoea batatas*) genome to pseudo-chromosomes based on synteny with close species^15^. Moreover, in both cases the N50 contig size was relatively low (45 and 5.6 kb, respectively).

Improvement of cultivated alfalfa might be accelerated if agronomically beneficial mutations, especially recessive ones, could be easily incorporated into modern varieties^16,17^. Natural or mutagen-induced mutations occur randomly and inefficiently, so obtaining mutants of the autotetraploid and self-incompatible cultivated alfalfa through traditional phenotypic selection is challenging. However, revolutionary site-specific CRISPR/Cas9 nuclease technology has been successfully applied for simultaneously editing multiple alleles and creating (precisely and predictably) mutants of various polyploid plants, such as hexaploid bread wheat and tetraploid durum^18^, allohexaploid *Camelina sativa*^19^, and allotetraploid cotton^20^. It also provides a feasible means to circumvent the inherent difficulties of introducing mutations into the autotetraploid cultivated alfalfa^21^, but no mutant of the species has been previously reported using either CRISPR/Cas9 or other site-specific nucleases.

Here, we apply PacBio CCS (circular consensus sequencing) and Hi-C (High-throughput chromosome conformation capture) technology to generate an allele-aware chromosome-level genome assembly for the cultivated alfalfa. An efficient CRISPR/Cas9-based genome editing protocol is also developed on the basis of this genome assembly, and used to create null mutants with clear phenotypes. Moreover, the mutated alleles and phenotypes can be stably inherited in a transgene-free manner, which may facilitate the commercial breeding of cultivated alfalfa.

## Results

### Assembly and annotation of the autotetraploid alfalfa

In total, 70 gigabases (Gb) of PacBio CCS long reads and approximately 126 Gb of Illumina short reads were obtained, using Sequel and HiSeq2000 platforms, respectively (Supplementary Tables 1 and 2). The Canu software package^22^ was used to initially assemble the cultivated alfalfa genome, yielding an initial contig set with N50 value of 459 Kb. The total length of this initial assembly was 3.15 Gb, consisting with the estimates of cultivated alfalfa genome size obtained using flow cytometry and *K*-mer based methods (2*n* = 4×, ~3 Gb and ~3.15 Gb, respectively) (Supplementary Figs. 1 and 2). The CCS long-reads and Illumina short-reads were mapped against the initial assembly to check the heterozygosity and reads depth distribution. We noticed that most 5 kb windows (98.2%) contain no identified SNPs, and the remaining 1.2% windows have an average heterozygosity close to 0.02%. The reads depth distribution of genomic regions also exhibits a similar pattern that most regions have an average depth of 22, and only 3.2% of 5 kb windows have a depth larger than 44 (Supplementary Fig. 3). These results indicate that the initial contig assemblies well resolved the haplotypes of the autotetraploid cultivated alfalfa.

**Fig. 1:**
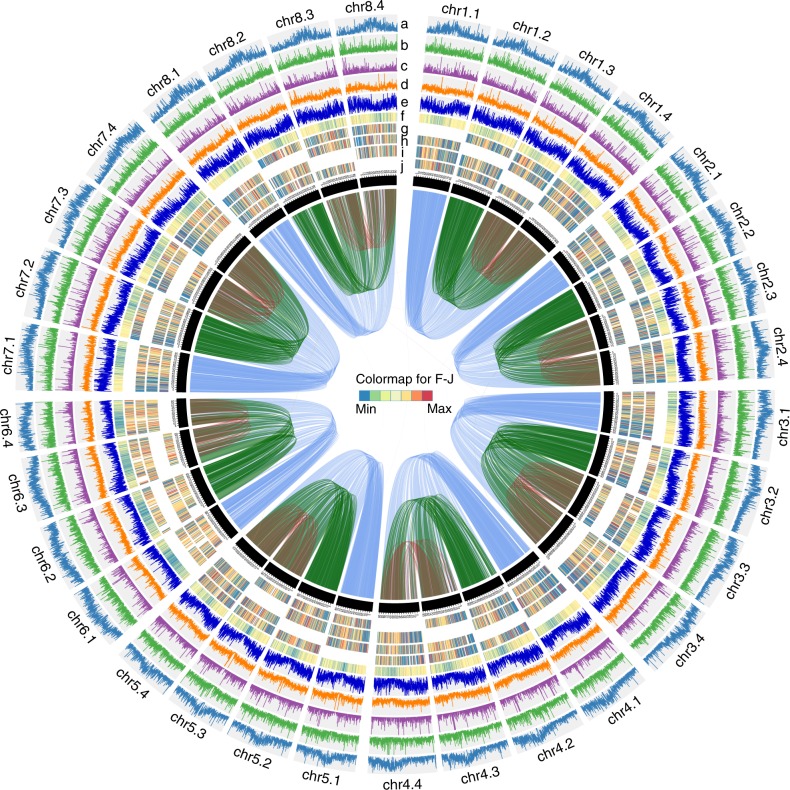
Overview of the cultivated alfalfa genome. The tracks indicate (moving inwards): **a** density of LTR transposons, **b** density of LINE transposons, **c** density of SINE transposons, **d** density of DNA transposons, **e** gene density, **f** gene expression levels, **g** *Ka/Ks* of syntenic gene pairs identified between chrX.1 and one of chrX.2, chrX.3, chrX.4, **h** *Ka/Ks* of syntenic gene pairs identified between chrX.2 and one of chrX.1, chrX.3, chrX.4, **i** *Ka/Ks* of syntenic gene pairs identified between chrX.3 and one of chrX.1, chrX.2, chrX.4, and **j** *Ka/Ks* of syntenic gene pairs identified between chrX.4 and one of chrX.1, chrX.2, chrX.3. Links in the core connect synteny blocks, blue ribbons indicate synteny blocks between chrX.1 and chrX.2, chrX.3, chrX.4, green ribbons indicate synteny blocks between chrX.2 and chrX.3, chrX.4, red ribbons indicate synteny blocks between chrX.3 and chrX.4. Source data are provided as a Source data file.

**Fig. 2:**
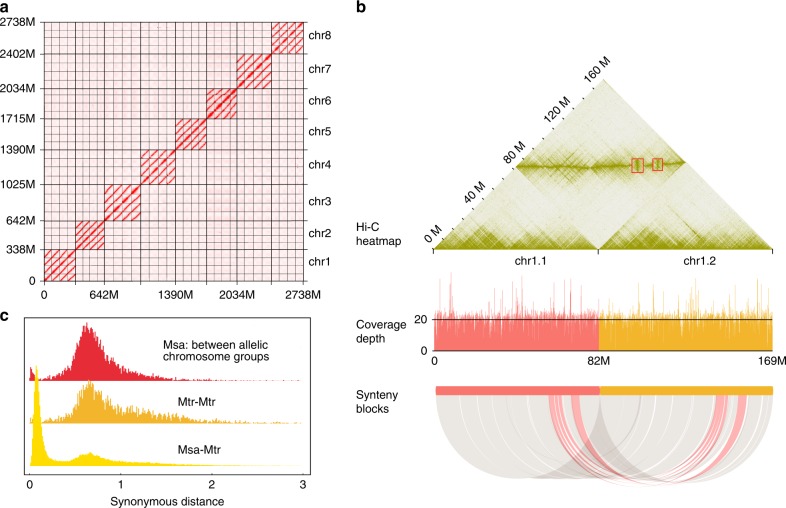
Assembly, similarity, and divergence of allelic chromosomes. **a** Overview of Hi-C heatmap for assembled chromosomes. Each allelic group contains four chromosomes, there are few linkages between allelic groups, indicating high quality chromosome-level scaffolding. **b** Chr1.1 and chr1.2 were chosen as examples to illustrate the assembly quality. The Hi-C heatmap shows the contiguity within chromosomes and the linkage between allelic chromosomes, the depth shows the even coverage of assembled sequence. The syntenic blocks show good synteny between chr1.1 and chr1.2, and two inversions were detected by both Hi-C heatmapping (red rectangles) and synteny (red ribbons). **c** Frequencies of synonymous distances between syntenic gene pairs, cross-comparing *M. sativa* (Msa) and *M. truncatula* (Mtr).

We next used the ALLHiC algorithm, which is capable of building allele-aware, chromosome-level assembly for autopolyploid genomes using Hi-C paired-end reads^14^, to scaffold the autotetraploid genome by integrating 1277 million read pairs of Hi-C data (Fig. 1, Supplementary Tables 3–6 and Supplementary Data 1). The final assembly contains 2.738 Gb in 32 super-scaffolds and 419 Mb of unplaced unitigs, representing all the 32 chromosomes comprising eight homologous groups with four allelic chromosomes in each. To validate the scaffolding of homologous group, we mapped a composite genetic linkage map of the cultivated alfalfa^23^ to our assembly and found the genetic map supports the chromosomal assignment (Supplementary Fig. 4). We further assessed the assembly quality by investigating the Hi-C contact matrix. The plotted Hi-C linkage shows that the chromosome groups are clear cut (Fig. 2a, b). We also sequenced 99 Gb ONT (Oxford Nanopore Technology) long reads with average reads length of 16 Kb (Supplementary Table 7). The top 200 longest ONT reads, ranged from 95 to 263 Kb were extracted and mapped against the chromosomes, and most of them (89%) could be mapped with one single chromosome with a length larger than 80% of its own length, indicating that most of the chromosomes were phased correctly. Accordingly, the four monoploid genomes (each consisting of eight chromosomes) contain 88.50, 88.30, 87.50, and 87.20% complete BUSCO genes, respectively, and a combined of 97.2% complete BUSCO genes as a whole (Supplementary Table 8). In addition, more than 90% of the assembled transcripts could be mapped to the genome (Supplementary Table 9). Based on the chromosome-level assembly, a total of 164,632 protein-coding genes were identified and more than 95.4% genes were functionally annotated via searches of NR, GO, KEGG, Swiss-Prot and TrEMBL databases (Supplementary Fig. 6, Supplementary Tables 10 and 11). Taken together, these results confirm the well-organized allele-aware chromosome-level assembly and gene annotation.

We found a low level of sequence divergence (~0.01) between any allelic chromosome pairs and the genetic linkage map could not distinguish homologous relationships (Supplementary Figs. 4 and 5), suggesting they have experienced abundant recombination, providing the molecular evidence for the conjecture that the cultivated alfalfa is a tetrasomic inherited autoploid plant in which bivalent pairing is random and not preferential^9,24^. Therefore, it is conceivable that the four allelic chromosomes are mostly functionally equivalent, like the two allelic chromosomes in diploid species. To validate this hypothesis, we compared the four allelic chromosomes systematically. The results showed that the four allelic chromosomes were highly similar in terms of size, number of genes and contents of repeat elements (Supplementary Data 2). Plots of the synteny relationship and *Ka/Ks* ratio of each syntenic gene pair, clearly show a high degree of conserved synteny, with no substantial overall *Ka/Ks* ratio difference between any two allelic chromosomes (Fig. 1). We also investigated expression levels of genes in each allelic chromosome group, and detected no significant overall allele dominance in the expression profiles of the cultivated alfalfa (Supplementary Fig. 7). All these results indicate that the autotetraploid alfalfa is a stable, random pairings autotetraploid species, unlike the more common situation of returning to diploid state accompanied with massive gene loss after whole genome duplication^25^. This situation has hindered deciphering genome all the time. Fortunately, based on the most optimal technology available to date (accurate CCS reads, Hi-C data, and allele-aware assembly algorithm), we successfully assembled all allelic chromosomes for one plant of cultivated alfalfa, although there may exist some errors in phasing the four allelic chromosomes due to its essential features of tetrasomic inheritance. In addition, we have to point out that to correctly phase four homologous chromosomes is only meaningful for individual plant in such tetrasomic and self-incompatible alfalfa, as the widespread recombination occurs in various cultivars and different individuals. Nevertheless, this well-organized chromosome-level assembly has sufficient quality for most genetic dissection and breeding research on cultivated alfalfa.

### Whole genome duplication and bursts of transposable element

We next inferred the phylogeny position and divergence times between cultivated alfalfa and another ten legume species and grape (*Vitis vinifera*). Here we selected the first group (chr1.1–chr1.8) to represent monoploid alfalfa in this analysis. In total, 569 single copy genes were identified and used to construct the phylogenetic relationships, via the concatenated and multispecies coalescent approach (Supplementary Fig. 8). The results indicate that cultivated alfalfa and *M. truncatula*, the most closely related species, diverged ~5.3 (3.7–7.3) million years ago (Mya). As complex polyploidization events occurred in ancestral or specific legume species^26–28^, we also used 980 conserved BUSCO genes and 5305 low copy genes (≤10 genes for each species) to infer the phylogeny, resulting in the same topology as that obtained using single copy genes (Supplementary Fig. 9). All the phylogeny analyses provided very high support (bootstrap values equal to 100% or posterior probability equal to 1) for each node, except the basal lineages *Arachis* and *Lupinus*. This may be due to the very recent divergence (~4.58 Mya) of the ancestral legume into *Arachis* and *Lupinus* (Supplementary Fig. 8), and incomplete lineage sorting and early gene flow may have influenced the robustness of this topology^29^.

The haploid size of the cultivated alfalfa genome (assembled 2738 Mb/4 = 685 Mb) is 295 Mb greater than that of the *M. truncatula* genome (published assembly is 390 Mb although the estimated size was 430 Mb^12^). We analyzed whole-genome duplication (WGD) events and the transposable element (TE) content in these allelic chromosomes, which have had profound effects on plant genome evolution^30^. Distributions of synonymous substitutions per synonymous site (*Ks*) within genes in syntenic blocks clearly indicated that the same ancient WGD occurred in the evolutionary history of all 11 genome-sequenced legume species (*Arachis duranensis*, *Cajanus cajan*, *Cicer arietinum*, *Glycine max*, *Lotus japonicus*, *Lupinus angustifolius*, *M. sativa*, *M. truncatula*, *Phaseolus vulgaris*, *Trifolium pretense* and *Vigna angularis*) compared in this study^31,32^ (Fig. 2c and Supplementary Fig. 10). The estimated date of this WGD (~58 Mya)^31,32^ and associated *Ks* values in the cultivated alfalfa genome (~0.63) indicated an average mutation rate (*μ*) of 5.43 × 10^−9^ per site per year in alfalfa, slightly lower than corresponding values in *M. truncatula* (*Ks* ~0.65, *μ* 5.60 × 10^−9^ per site per year) (Fig. 2c).

TEs account for 55% of the assembled cultivated alfalfa genome (Supplementary Table 12 and Supplementary Data 2). Long terminal repeat (LTR) retrotransposons are the most abundant TEs (account for 27.36% of the genome), and more abundant in the cultivated alfalfa genome than in *M. truncatula* (13.37%) (Supplementary Table 12). Among the LTR retrotransposons, the Ty3/Gypsy superfamily is more abundant than the Ty1/Copia superfamily in both cultivated alfalfa and *M. truncatula* (Supplementary Table 12 and Supplementary Data 2). The comparison of genomic contents of cultivated alfalfa and *M. truncatula* shows that Ty3/Gypsy elements contribute most to the inflated cultivated alfalfa genome, accounting for 31.93% ((123 – 29 Mb)/295 Mb) of its genome size increment. Non-repeat sequences, Ty1/copia, simple/tandem repeat, and DNA element account for 26.57, 13.56, 9.93, and 6.87% of the total difference, respectively (Supplementary Table 12). We also found that LTR bursts occurred in both species recently (<2 Mya), after the two species diverged (Supplementary Fig. 11), but stronger in the cultivated alfalfa. Collectively, these results show that accumulation of TE insertions was the main reason for the enlarged cultivated alfalfa’s genome.

### Establishment of the CRISPR/Cas9 genome editing protocol

**Fig. 3:**
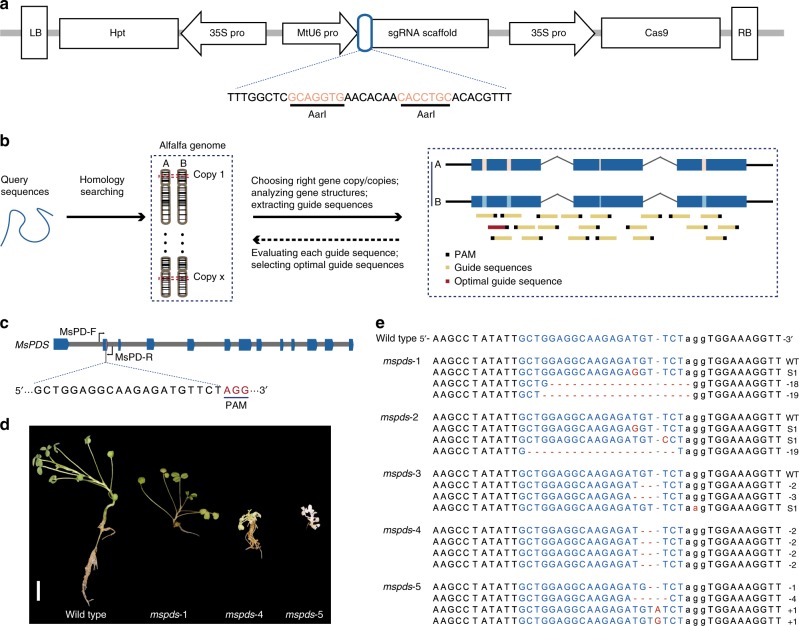
CRISPR/Cas9-mediated genome editing in autotetraploid cultivated alfalfa. **a** Schematic illustration of the Cas9 and sgRNA expression cassettes in the pMS-CRISPR/Cas9 vector (LB left border, RB right border, genes are represented by rectangles and promoters by arrows). The sgRNA consists of a guide sequence (blue rounded rectangle) and a scaffold, and the region between two AarI restriction endonuclease sites (light red) is used for the ligation of guide sequences. **b** Pipeline for designing guide sequences based on the cultivated alfalfa genome. **c** Guide sequence for *MsPDS*. Dark blue boxes and gray lines represent exons and introns, respectively. PAM is shown in red. **d**, **e** Genome editing of *MsPDS*. **d** Photos of three representative *MsPDS* mutants (*mspds*-1, *mspds*-4, and *mspds*-5), with *mspds*-1 exhibiting a wild type phenotype, and *mspds*-4 and *mspds*-5 exhibiting dwarf, albino phenotypes. Scale bar, 1 cm. **e** Sequencing of all screened mutants confirmed the presence of mutations at the target sites (light blue). The PAM regions are shown in black and lowercase. Nucleotide deletions, insertions or substitutions are shown in red, with details given in the right panel.

The allele-aware chromosome-level cultivated alfalfa genome assembly obtained in this study provides a necessary start point to accurately apply the CRISPR/Cas9 technology to help in screening candidate genes, decoding gene structural information and designing optimal guide sequences (Fig. 3b, detailed in “Methods”). Conversely, this genome editing technology could help efforts to convert the enormous amount of genome data into functionally relevant knowledge. A plant transformation binary vector named pMs-CRISPR/Cas9 (Fig. 3a) was constructed to stably transform alfalfa cultivars using *Agrobacterium tumefaciens* (Supplementary Fig. 12). In this vector, the CaMV 35S promoter is used to express *hSpCas9*^33^ and the selectable marker gene *Hygromycin phosphotransferase* (*Hpt*), and the MtU6 polymerase III promoter^34^ is used to drive expression of sgRNAs.

The *Phytoene desaturase* (*PDS*) gene was selected for the first test of this CRISPR/Cas9 system’s efficacy, as null *pds* mutants generally have clearly visible albino and dwarf phenotypes during juvenile stages^35^. Four nearly identical *MsPDS* alleles were identified by analyzing the alfalfa genome assembly and manually checking (Supplementary Fig. 13). A guide sequence located in the conserved region in exon 2 of *MsPDS* (Fig. 3c) was chosen, and was then synthesized and integrated into the pMs-CRISPR/Cas9 vector. After transformation, 50 plants were regenerated from 880 transformed calli, two of which (designated *mspds*-4 and *mspds*-5) exhibited the anticipated albino and dwarf phenotypes (Fig. 3d). All regenerated plants were initially screened for mutations by directly sequencing PCR amplicons encompassing the target site, and the mutagenesis frequency was defined as the number of mutants divided by the total number of transformed calli^18^. Sequencing chromatograms indicated that five plants were mutants (5/880, 0.57%, designated as *mspds*1 to 5) (Supplementary Fig. 14). To further confirm the CRISPR/Cas9-induced *mspds* mutants and directly validate the sequencing results, PCR amplicons of *MsPDS* from candidate mutants were sub-cloned and 30 positive recombinant clones were randomly sequenced. This confirmed that all five screened mutants had mutated alleles at the target site. In detail, *mspds*-1, *mspds*-2, and *mspds*-3 contained three mutated alleles and one wild-type allele, while *mspds*-4 and *mspds*-5 had mutations in all four alleles (0.23%) (Fig. 3e). Due to the presence of a wild-type *MsPDS* allele, *mspds*-1, *mspds*-2, and *mspds*-3 plants displayed a wild type phenotype, whereas *mspds*-4 and *mspds*-5 plants displayed dwarf and albino phenotypes. The results of editing *MsPDS* demonstrate that the developed CRISPR/Cas9 system can be used for introducing mutations into the cultivated alfalfa genome. Importantly, null mutants can be created in the T0 generation.

### Transgene-free and stably inherited mutations of *MsPALM1*

**Fig. 4:**
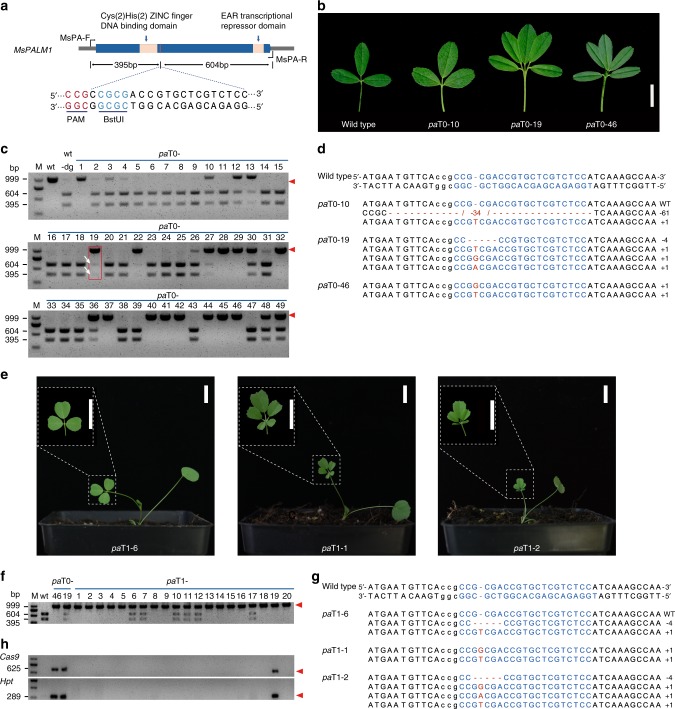
Genome editing of *MsPALM1*, and generating transgene-free and stably inherited *palm1*-type progenies. **a** A guide sequence for *MsPALM1*. Dark blue box represents exon. PAM is shown in red. The BstUІ site is underlined and shown in light blue. **b** Leaf morphologies of three representative T0 plants. Scale bar, 1 cm. **c** Results of PCR-RE analyses for identifying mutants among T0 plants. In the gel, wt and wt-dg lanes contain DNA samples from wild-type plants without and with digestion by the BstUІ restriction endonuclease, respectively. Red arrowheads indicate bands used to identify mutations. Notably, *pa*T0*-*19 (highlighted with a red rectangle) yields dim digested bands (indicated by white arrows), although it develops palmate-like pentafoliate leaves (**b**). **d** Genotyping of the corresponding mutants in **b** confirmed the presence of mutations at the target sites (light blue). The PAM regions are shown in black and lowercase. Nucleotide deletions, insertions, or substitutions are shown in red, with details in the right panel. **e** Three representative T1 plants with two showing anticipated *palm1*-type leaf morphologies like their parents. Scale bar, 1 cm. **f**, **g** Results of PCR-RE analyses (**f**) and sequencing analyses (**g**) confirmed the parental *MsPALM1* mutations in corresponding T1 progenies in **e**. **h** Outcome of tests for transgene-free mutants in 20 corresponding T1 progenies in **f**. Lanes without bands (indicated by red arrowheads) identify transgene-free mutants. Lanes labeled wt, *pa*T0-19 and *pa*T0-46 show PCR fragments amplified from a WT plant, and two T0 mutants (*pa*T0-19 and *pa*T0-46), respectively. Source data underlying Fig. 4c, f, h are provided as a Source Data file.

A high leaf/stem ratio is an important agronomic trait for cultivated alfalfa, as it is positively correlated to the nutritional value of alfalfa products^6^. Breeding varieties with more leaflets per leaf may improve the leaf/stem ratio of cultivated alfalfa and thus increase its yield and nutritional value. In diploid *M. truncatula*, *PALM1* encodes a Cys(2)His(2) zinc finger transcription factor that plays a key role in compound leaf morphogenesis. Null *palm1* mutants develop palmate-like pentafoliate leaves rather than wild-type trifoliate leaves^36^. Thus, we hypothesized that disruption of *PALM1* orthologs (*MsPALM1*) in cultivated alfalfa may enable it to express the *palm1* phenotype. This would also provide another easily visible example to validate the stability of our protocol and its potential for generating multileaflet varieties. Four *MsPALM1* alleles were identified and all *MsPALM1* copies were found to have a single exon (Supplementary Fig. 15). To disrupt *MsPALM1*, we selected a specific guide sequence to guide Cas9 to disrupt a BstUI restriction endonuclease site, thereby enabling easy screening of mutants through PCR-Restriction enzyme (PCR-RE) assay (Fig. 4a).

In total, we identified 26 mutants from 1508 transformed calli (1.72%), including 12 *palm1*-type plants (0.80%) that developed palmate-like pentafoliate leaves (Fig. 4b, c). Sanger sequencing of 20 clones of each mutant confirmed the presence of at least one mutated *MsPALM1* allele in their genomes, and all four alleles were disrupted in the *palm1*-type plants (Fig. 4d and Supplementary Fig. 16). Notably, three *palm1*-type plants (*pa*T0-1, *pa*T0-19, and 29) were identified as chimeric mutants. Although the leaf morphology and sequencing results (Fig. 4b, d) show that all four *MsPALM1* alleles in *pa*T0-19 were mutated, dim digested bands were still detected in PCR-RE analysis of this mutant (Fig. 4c), indicating that wild-type alleles may persist in some of its cells. Furthermore, *pa*T0-1 and -29 contain up to five mutation types (Supplementary Fig. 16), indicating that their cellular genotypes are not uniform, as reported in other T0 CRISPR/Cas9-edited plants^37,38^. To comprehensively investigate the off-target effects, whole genomes of three *palm1*-type mutants (*pa*T0-1, *pa*T0-19, and *pa*T0-46) were resequenced with 30-fold depth using IIlumina sequencing technology. Global scanning of these mutants’ whole genomes detected no off-target mutations in protein-coding regions besides the targeted regions (Supplementary Table 14). This demonstrates that off-target effects of mutating cultivated alfalfa can be largely eliminated by using the developed CRISPR/Cas9-based genome editing protocol with the guidance from our high-quality genome.

Stable inheritance of agronomically beneficial mutations in cultivated alfalfa is hindered by its polyploidy and cross-pollination. To investigate whether the mutations and phenotypes of *palm1*-type mutants could be transmitted to the next generation, we harvested T1 seeds from *pa*T0-19 and *pa*T0-46 crosses (Supplementary Fig. 17). Twenty seeds were randomly chosen and sown in a greenhouse, and 14 of the plants that germinated from them were *palm1*-type plants (Fig. 4e). PCR-RE and sequencing analyses confirmed that each of these 14 *palm1*-type offspring contained four mutated *MsPALM1* alleles originating from their parents. Each of the six plants with wild-type phenotypes had at least one unmutated allele (Fig. 4f, g and Supplementary Fig. 18), very possibly resulting from chimeric effects in *pa*T0-19. We also detected transgene-free plants by PCR analysis with two primers specific for *hSpCas9* and *Hpt* (Supplementary Table 15). The T-DNA fragment was found to be absent in 13 *palm1*-type progenies (Fig. 4h). Collectively, these results show that our CRISPR/Cas9-based genome editing protocol can rapidly introduce heritable mutations and phenotypes into cultivated alfalfa in a transgene-free manner. In addition, the generation of these transgene-free *palm1-*type progenies indicates that CRISPR/Cas9 technology may provide a shortcut for breeding multileaflet varieties which may have higher nutritional value, although further studies are required to test whether the increase in leaflet number is accompanied by improvements in leaf biomass and forage quality.

## Discussion

This study provides two complementary contributions, the chromosome-level reference genome and CRISPR/Cas9-based genome editing protocol, with substantial potential for accelerating fundamental investigation and breeding of cultivated alfalfa. In summary, by exploiting new sequencing technology and Hi-C scaffolding, we are able to decode the complex autotetraploid cultivated alfalfa genome, reveal events that have apparently shaped it, and create foundations for further studies on legumes and complex genome assembly. The genome is also a valuable resource for studies of alfalfa biology, evolution, and genome-wide mapping of QTLs associated with agronomically relevant traits. Due to its tetrasomy and self-incompatibility, improvement of cultivated alfalfa through traditional breeding approaches requires long breeding cycles and screening of extremely large populations in order to accumulate randomly occurring natural or mutagen-induced mutations conferring desirable traits at high frequencies. By contrast, using our genome assembly, we establish a reliable CRISPR/Cas9-based genome editing protocol for cultivated alfalfa that can precisely and simultaneously disrupt all alleles of selected genes (here, *MsPDS* and *MsPALM1*), thereby creating null mutants in a single generation. Most importantly, the mutated alleles and phenotypes can be stably transmitted to progenies by cross-pollination between two mutants in a transgene-free manner, which may help to accelerate the breeding speed and mitigate concerns about transgene technology and its products. The results also provide robust foundations for further technical developments, such as precise knock-in, base editing, or regulation of expression. Thus, they could potentially raise global food security by reducing breeding periods and costs of improving key agronomic traits of this important crop.

## Methods

### Sources and sequencing of genomic DNA/RNA

Fresh leaves were plucked from a single cultivated alfalfa (cultivar XinJiangDaYe) plant cultivated in a greenhouse kept at 21–23 °C, 16 h light per day (light intensity of 380–450 W per m^2^) and a relative humidity (RH) of 70%. DNA was extracted from these leaves using a DNeasy Plant Mini Kit (Qiagen). Portions of the DNA were sent to AnnoRoad (Ningbo, China) to construct circular consensus sequencing (CCS) libraries and sequence them using a PacBio Sequal platform, and other portions were sent to Nextomics (Wuhan, China) to construct libraries and sequence them using Nanopore ONT and Illumina Hiseq platforms. These sequencing efforts yielded 70, 99, and 126 Gb of reads, respectively, for de novo assembly of the cultivated alfalfa genome (Supplementary Tables 1 and 2).

In addition, tender roots and shoots with leaves were collected, and RNA was extracted from one pooled root and shoot sample (with roughly the same weight of each organ) and four leaf samples using an RNeasy Plant Mini Kit (Qiagen). RNA samples were reverse-transcribed using random primers and sequenced using an Illumina platform. The RNA-seq data obtained are summarized in Supplementary Table 9.

### Hi-C library construction and sequencing

Fresh leaves and shoots were plucked from the plant used for whole genome sequencing, and then chromatin in the samples was cross-linked to DNA and fixed^39^. Fixed samples were sent to BGI-Qingdao (Qingdao, China) for Hi-C library construction and sequencing. Two libraries were constructed using DpnII restriction endonuclease and 200 Gbp of data were obtained (Supplementary Table 3).

### Genome size estimation

Illumina data were cleaned using Trimmomatic (v. 0.36)^40^ with default parameters. Two libraries (lib3 and lib4 in Supplementary Table 1), each with about 56 Gbp of reads, were used to estimate the cultivated alfalfa genome size by *K*-mer (*K* = 17) frequency-based methods with Kmerfreq in the SOAPec (v. 2.01) package^41^. The estimated genome size was 1,578,294,649 bp, based on a frequency peak near 38× (Supplementary Fig. 1), in accordance with previous findings^42^. Two other visible peaks near 20× and 70× reflect the heterozygosity associated with out-crossing and repetitive nature of auto-polyploid genomes. The heterozygosity rate is 3.7%, according to estimates obtained using a homemade script. The genome size of the sequenced individual was confirmed by flow cytometry^42^ (Supplementary Fig. 2), as follows. Leaves from *M. sativa* (cultivar XinJiangDaYe) and *M. truncatula* (cultivar Jemalong, A17) plants were finely chopped together with a razor blade in 400 μl Galbraith buffer with 5 μl ml^−1^ β-mercaptoethanol. The resulting suspension was filtered through 30-μm nylon. From a 500 U ml^−1^ stock of Ribonuclease A, was added, from a 500 U ml^−1^ stock solution, to 10 μl ml^−1^ and propidium iodide to 50 μg ml^−1^. After 30 min incubation at room temperature, the DNA peak ratio was assessed by flow cytometry.

### Genome assembly

The cultivated alfalfa genome was assembled as follows: (1) We assembled contigs from CCS clean reads using Canu^22^, with default parameters. The N50 values of the contig sets were 459 kb, with total lengths 3154 Mb. (2) Hi-C reads were aligned to contigs using HiC-Pro^43^, yielding an alignment BAM file. (3) Contigs were annotated with a solely homology-based strategy, using annotated *Medicago truncatula* proteins as references. 138,729 homologous genes were structurally annotated. MCscan in Jcvi (https://zenodo.org/record/31631#.XpkUyTOeask) was used to identify synteny blocks between contigs and the reference genome. Contigs syntenic to *M. truncatula* were stacked and aligned to *M. truncatula* chromosomes. The syntenic contigs are summarized in Supplementary Table 4. (4) An in-house script was used to prune the BAM file and discard links between allelic contigs. Contigs syntenic to one chromosome of *M. truncatula*, e.g., chr1, were extracted, sub-clustered and reordered using ALLHiC^44^, yielding a raw scaffold set. (5) Juicebox^45^ was used for fine-tuning assembled scaffolds in a graphic and inter-active fashion. Forty scaffolds with a total length of 1800 Mb were cropped (Supplementary Table 5). (6) Based on this scaffold assembly, each unplaced contig was assigned to the contig cluster, to which the contig was most connected by Hi-C data. (7) Those contig clusters were reordered and scaffolded using ALLHiC. (8) Using Juicebox, scaffolds were fine-tuned and discordant contigs were removed from scaffolds, and the final chromosome assembly was generated, containing 32 chromosomes with a total length of 2738 Mb (Supplementary Table 6).

### Genome annotation

Repetitive sequences of the cultivated alfalfa genome were annotated using both homology-based search and ab initio methods. TRF (v. 4.07b)^46^ was used to identify tandem repeats. RepeatProteinMask and RepeatMasker (v. 4.0.5) were utilized to search for known transposons (RepeatMasker using a library built by RepeatModeler) and LTR_FINDER was used for ab initio repeat identification (Supplementary Table 12).

All repetitive regions except tandem repeats were soft-masked for protein-coding gene annotation. The coding sequences of *Arabidopsis thaliana*, *V. vinifera*, *G. max*, *Oryza sativa*, *M. truncatula*, *Cicer arietinum* and *Lotus japonicus* were downloaded. These coding sequences were subjected to Blast (v. 2.2.26) searches against the cultivated alfalfa genome and alignments were extracted for structural inspection by GeneWise in the Wise2 package (v. 2.2.0)^47^. Homologs containing premature stop codons and frameshifts were discarded. Ms-root-shoot RNA-seq data were aligned to alfalfa contigs using Blat (v. 34)^48^ and GMAP (v. 2016-11-07)^49^, and a comprehensive transcriptome database was built using PASA (v. 2.2.0)^50^. Open reading frames (ORFs) were predicted using TransDecoder, and the resulting database was used to train parameters for the following four de novo gene prediction software packages: AUGUSTUS (v. 3.2.2)^51^, GeneID (v. 1.4.4)^52^, GlimmerHMM (v. 3.0.2)^53^, and SNAP (v. 2006-07-28). Predictions obtained using these packages were then combined using EVM (v. 2012-06-25)^54^ (Supplementary Fig. 6), then 87,479 protein-coding genes were retrieved and functionally annotated by blast searches against databases including UniProtKB/Swiss-Prot and UniProtKB/TrEMBL (last accessed on Sep 17th, 2014)^55^. They were also subjected to GO annotation and protein family annotation by InterProScan (v. 5.17-56.0)^56^. KO terms for each gene were assigned by blast searches against the KO database (last accessed on Sep 10th, 2014) (Supplementary Table 11).

### Genome synteny

MCScanX^57^ was employed to identify syntenic blocks in alfalfa and *M. truncatula*. Pairwise *Ks* values of syntenic paralogous genes were estimated by the “add-ka-and-ks-to-collinearity” program in MCscanX software, with Nei-Gojobori statistics. *Ks* values for each syntenic gene pair were then calculated with an in-house Perl script available at https://github.com/stanleyouth/-/blob/master/synteny_dn_ds.pl.

### Phylogenetic analysis

All protein sequences from 11 species (*Arachis duranensis*, *Cajanus cajan*, *Cicer arietinum*, *G. max*, *Lotus japonica*, *Lupinus angustifolius*, *M. truncatula*, *P. vulgaris*, *T. pretense*, *Vigna angularis*, and *Vitis vinifera*) obtained from the NCBI database were used to generate clusters of gene families. As the allelic chromosomes have highly similar gene contents, the first allelic chromosome group (chr1.1–chr1.8) was selected to represent monoploid alfalfa. Gene sets were filtered by selecting the longest ORF for each gene. ORFs with premature stop codons, that were not multiples of three nucleotides long, or encoded less than 50 amino acids, were removed. Orthologous genes were identified by OrthoMCL. Single-copy genes (569) were identified, and subsequently used to build a phylogenetic tree. Coding DNA sequence (CDS) alignments of each single-copy family were created based on the protein alignment, using MUSCLE software^58^. A phylogenetic tree was reconstructed with RAxML software^59^ under the GTR+ gamma model with each single-copy gene and the concatenated sequence. ASTRAL^60^ was used to construct a coalescent tree from the gene trees. We also extracted the most complete sequence for each BUSCO gene in each species, and then concatenated all the shared 980 single-copy BUSCO genes for tree building with the same method. Finally, the low copy gene-based (LCG) method^61^ was applied to avoid the limitations of single copy genes, using a total of 5305 LCGs shared among the 12 species with less than ten copies in each species. The gene family trees were constructed and STAG^62^ software was used to infer the species trees. To estimate divergence times, we used the PAML mcmctree program^63^ for approximate likelihood calculations, with the single copy genes identified by OrthoMCL, a correlated molecular clock model and a REV substitution model. After a burn-in of 5,000,000 iterations, the MCMC process was repeated 20,000 times with a sample frequency of 5000. Convergence was checked by Tracer v. 1.4 (http://beast.community/tracer) and confirmed by two independent runs. Two constraints were applied in time calibrations: 105–115 Mya for the *V. vinifera*—leguminous split, and 49–62 Mya for the *Arachis duranensis*—other leguminous species split.

Target gene analysis and guide sequence design: To locate and clone candidate genes, homologs of query sequences (such as known CDSs of candidate genes or orthologs from other organisms) were sought by alignment with the alfalfa genome. After mapping whole genome sequencing (WGS) reads to the corresponding CDS, information of the candidate genes, such as copy number and gene structures, was deciphered and computational results were verified by experimental examination. Then, a series of guide sequences were extracted from selected genes using home-made scripts (https://github.com/stanleyouth/-/blob/master/crispr.sgRNA.finder.pl). Guide sequences located in conserved coding exons were evaluated for potential off-target sites flanking protospacer adjacent motifs (PAMs: NGG and NAG) in the alfalfa genome using sgRNAcas9 (v. 3.0.5)^64^, which allows off-target sites with no more than 5 nt mismatches. Guide sequences were chosen that: covered all alleles; had no obvious off-target sites; were close to a start codon or in a functional conserved domain; had high GC content (which correlates with sgRNA efficacy); and started with a G at the 5′ end (required by the vector).

In this study, the mRNA sequence of the *MtPDS* gene of *M. truncatula* (accession code: XM_024777859.1) and CDS of the *MsPALM1* gene of *M. sativa* (accession code: HM038483.1) were used as query sequences to search and decode information on *MsPDS* and *MsPALM1*, respectively. Both *MsPDS* and *MsPALM1* were confirmed to have four alleles in the cultivated alfalfa genome. Series of guide sequences were extracted from exons of *MsPDS* and the single exon of *MsPALM1* (Supplementary Data 3 and 4). A previously used guide sequence in *M. truncatula* (5′-GCTGGAGGCAAGAGATGTTCT-3′)^34^, located in the conserved region in exon 2 of *MsPDS*, was inspected. It covers all *MsPDS* alleles and has no obvious off-target-site according to previous studies^65^ (Supplementary Data 3). The best target (5′-GGAGACGAGCACGGTCGCGGCGG-3′), which contains a BstUI restriction endonuclease site that overlaps with the predicted cleavage site for the Cas9/sgRNA complex (Fig. 3c), was screened for *MsPALM1*. No obvious off-target site was found for this guide sequence (Supplementary Data 4).

### Construction of CRISPR/Cas9 binary vector

The pMs-CRISPR/Cas9 vector was assembled by combining the expression cassettes of *hSpCas9* and sgRNA into the pCambia1300 entry vector, which contains a *Hpt* expression cassette. Pairs of oligos including guide sequences were synthesized as primers, annealed and cloned into AarI-digested pMs-CRISPR/Cas9 with T4 DNA ligase (NEB, Beijing, China). The pMs-CRISPR/Cas9 vector containing the guide sequence was transformed into competent *Escherichia coli* DH5α cells. Colony sequencing was used to confirm the correct insertion with Vt-F (Supplementary Table 15). A single colony was then propagated by cultivation in liquid LB medium containing 50 mg l^−1^ kanamycin, and the plasmid DNA was extracted using a TIANprep Mini Plasmid Kit (Tiangen, China) according to the manufacturer’s instructions. After that, plasmids of various CRISPR/Cas9 constructs were transferred into *Agrobacterium tumifaciens* strain EHA105 via electroporation for plant transformation experiments.

### Plant materials, growth, and generation of transgenic plants

Plants of the cultivated alfalfa cultivar Aohan (other cultivars were also used, unpublished data) were used as hosts for *Agrobacterium*-mediated transformation^66^ with some modification. Briefly, surface-sterilized seeds were sown on MS semi-solid medium and grown under long-day (16 h light/8 h dark) conditions at 25 °C. Fully developed cotyledonary explants from 7-day-old seedlings were excised and placed in Callus Induction Medium (SH basal salts and vitamins, 0.2 mg l^−1^ kinetin, 2 mg l^−1^ 2,4-D, 0.3 mg l^−1^ casein hydrolysate, 30 g l^−1^ sucrose, 8 g l^−1^ agar, pH 5.8). *Agrobacterium tumefaciens* strain EHA105 carrying binary vector was used to transform calli, as follows. A suspension of the strain was prepared in MSH liquid medium containing 100 μM acetosyringone, 0.025 mg l^−1^ kinetin and 2 mg l^−1^ 2,4-D. Calli were then submerged in the suspension in a covered conical flask, placed on a shaker and rotated at 75 rpm at room temperature for 10 min. The calli were placed on sterilized filter paper in a Petri dish, then transferred to Co-incubation Medium (MS basal salts and vitamins, 2 mg l^−1^ 2,4-D, 0.2 mg l^−1^ kinetin, 100 μM acetosyringone, 30 g l^−1^ sucrose, 8 g l^−1^ agar, pH 5.8) in a growth chamber at 27 °C in the dark for 3 days. They were subsequently transferred onto Selection Medium (SH basal salts and vitamins, 0.2 mg l^−1^ kinetin, 2 mg l^−1^ 2,4-D, 250 mg l^−1^ cefotaxime, 15 mg l^−1^ hygromycin, 30 g l^−1^ sucrose, 8 g l^−1^ agar, pH 5.8) for 45 days. After selection cultivation, all calli were transferred to Shoot Induction Medium (SH basal salts and vitamins, 2 g l^−1^ casein hydrolysate, 0.4 mg l^−1^ kinetin, 250 mg l^−1^ cefotaxime, 5 mg l^−1^ hygromycin, 30 g l^−1^ sucrose, 8 g l^−1^ agar, pH 5.8) for more than 30 days, then regenerated shoots were transferred to Root Induction Medium (MS basal salts and vitamins, 250 mg l^−1^ cefotaxime, 1 mg l^−1^ IBA, 30 g l^−1^ sucrose, 8 g l^−1^ agar, pH 5.8). Finally, regenerated plants were transferred to soil and grown to maturity in a greenhouse.

### Detecting mutations by PCR-RE assay and Sanger sequencing

Genomic DNA was extracted from alfalfa leaf tissues using a DNA quick Plant System (Tiangen, China) according to the manufacturer’s instructions. Genomic regions surrounding the *MsPDS* and *MsPALM1* target sites were amplified by PCR with gene-specific primers (Supplementary Table 15). For *MsPDS* gene, PCR products of individual plants were directly sequenced for screening mutants, and mutations were confirmed by sequencing 30 clones after cloning the PCR amplicons into the T vector pMD^TM^ 19 (Takara, Japan). For the *MsPALM1* gene, PCR products were digested with BstUI restriction endonuclease (NEB, Beijing, China) according to the manufacturer’s instructions and the products were visualized by agarose gel electrophoresis. PCR amplicons of each mutant were cloned into the T vector pMD^TM^ 19, and 20 clones were randomly picked for Sanger sequencing.

### Analysis of off-target effects

Three *palm1*-type mutants (*pa*T0-1, *pa*T0-19, and *pa*T0-46) were chosen for sequencing by an Illumina Hiseq 2500 platform, and a total of 97.4 Gb raw data were produced (Supplementary Table 13). FastQC (https://www.bioinformatics.babraham.ac.uk/projects/fastqc/) was used to evaluate the quality of the raw reads, then Trimmomatic (0.36)^40^ was used, with default parameters, to exclude low quality reads. After that, the cleaned reads were mapped to the reference genome of cultivated alfalfa with Bwa (v. 0.7.12)^67^. Then the sam files were converted to bam files and duplicated reads were removed with Picard tools (v1.119, https://broadinstitute.github.io/picard/). Finally, the mutations were identified with suggested commands of the Genome Analysis Toolkit (GATK v. 3.5)^68^. After excluding the low quality mutations with suggested parameters, functional effects of the mutations were annotated with SnpEff^69^. These putative off-target mutations were manually examined to confirm whether they were indeed mutations and whether they were the targets themselves. According to previous report^70^, single nucleotide variations (SNVs) were excluded and only indel events at or near the −3 position relative to the PAM sequence were considered as Cas9-induced off-target mutations.

### PCR analysis for screening transgene-free progenies

Genomic DNA was extracted from leaf tissues of T0 parents and T1 progenies as mentioned above, and then subjected to PCR using primers listed in Supplementary Table 15.

### Downloaded data

*A. thaliana*, *V. vinifera*, *G. max*, *O. sativa*, and *M. truncatula* genome annotation files were downloaded from ftp://ftp.ensemblgenomes.org/pub/plants/release-39, *Cicer arietinum* genome and annotation files from ftp://parrot.genomics.cn/gigadb/pub/10.5524/100001_101000/100076, and *Lotus japonica* genome and annotation files from ftp://ftp.kazusa.or.jp/pub/lotus/lotus_r1.0.

### Reporting summary

Further information on research design is available in the Nature Research Reporting Summary linked to this article.

## Supplementary information

Supplementary Information

Peer Review File

Reporting Summary

Description of Additional Supplementary Files

Supplementary Data 1

Supplementary Data 2

Supplementary Data 3

Supplementary Data 4

## Data Availability

Data supporting the findings of this work are available within the paper and its Supplementary Information files. A reporting summary for this article is available as a Supplementary Information file. The datasets generated and analyzed during the current study are available from the corresponding author upon request. All genome and transcriptome sequencing raw data described in this article are publicly available in the NCBI database under project PRJNA540215, and the genome assembly files are available at https://figshare.com/projects/whole_genome_sequencing_and_assembly_of_Medicago_sativa/66380. The source data underlying Figs. 1, 4c, f, h are provided as a Source data file.

## Source data

Source Data

## Code availability

In-house perl scripts used in this study were deposited in Github, including scripts used for calculating *Ka*/*Ks* for synteny blocks [https://github.com/stanleyouth/-/blob/master/synteny_dn_ds.pl], and scripts used for sgRNA designing [https://github.com/stanleyouth/-/blob/master/crispr.sgRNA.finder.pl].
