# Supplementary material for: Allele-aware chromosome-level genome assembly and efficient transgene-free genome editing for the autotetraploid cultivated alfalfa
Source: Nat Commun. 2020 May 19;11:2494. doi: 10.1038/s41467-020-16338-x (PMC7237683; doi:10.1038/s41467-020-16338-x)
Supplement: Supplementary file 1 — Supplementary Information [file 41467_2020_16338_MOESM1_ESM.pdf]

Allele-aware chromosome-level genome assembly and efficient transgene-free genome editing for the autotetraploid cultivated alfalfa

Chen *et al.*

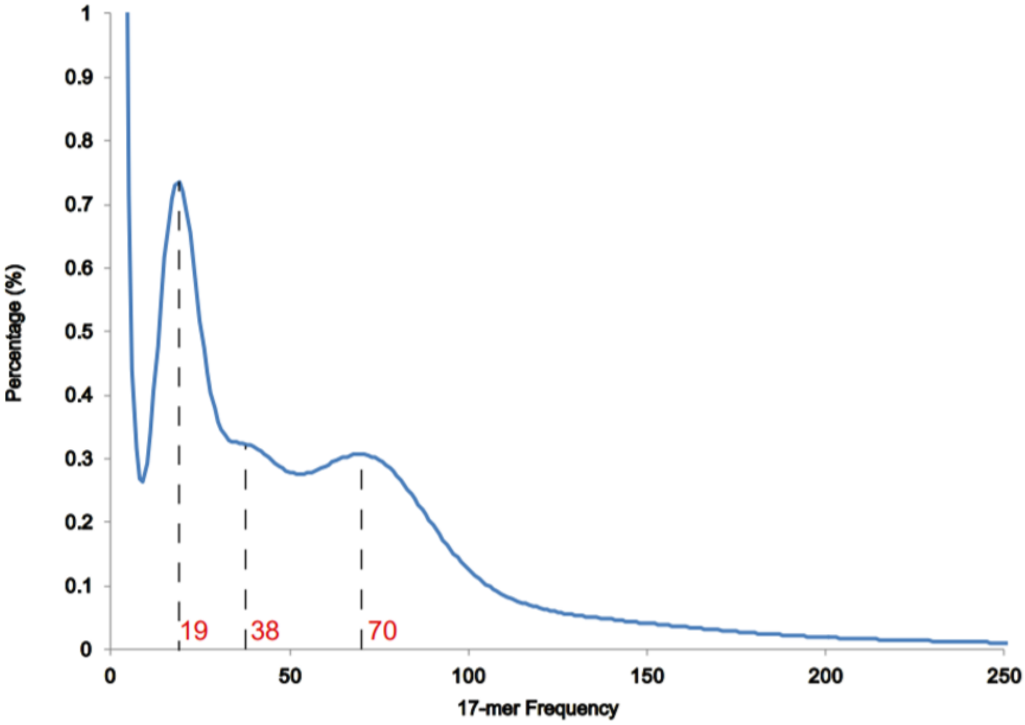

Supplementary Figure 1. Results of 17-mer frequency analysis to estimate the alfalfa genome size. The genome size was estimated using the frequency peak at 19 as coverage-depth. The peak at 19 is attributed to high heterozygosity, and the peak at 70 to autotetraploidy. The genome size was calculated by dividing the total *K*-mer count by coverage-depth (59,975,196,680 / 19 = 3,156,589,298).

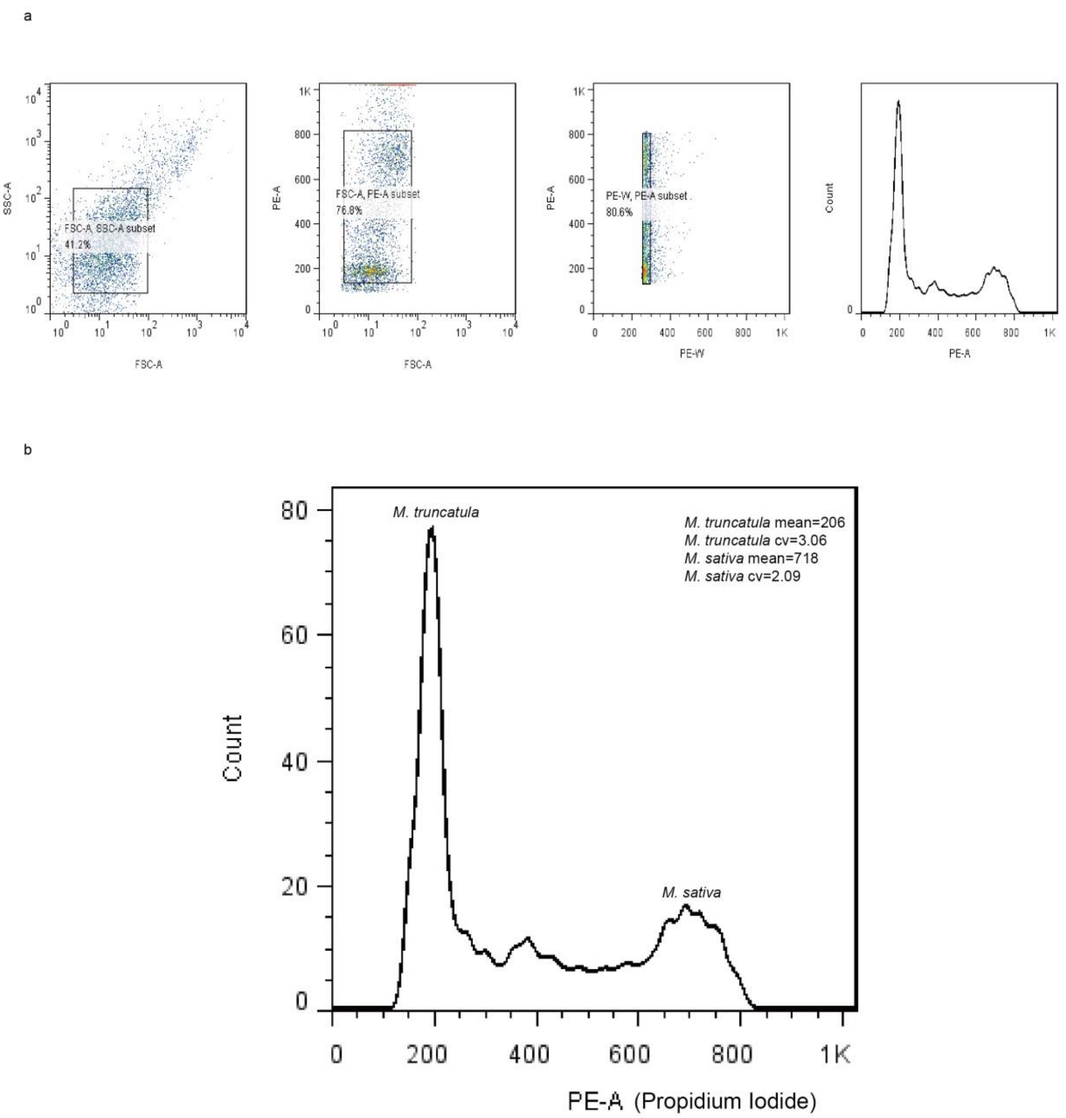

Supplementary Figure 2. Using flow cytometry to estimate genome size of cultivated alfalfa. a. The gating strategy. b. Results of flow cytometry. Histogram of relative fluorescence intensities from flow cytometric analysis of PI-stained nuclei of cultivated alfalfa and *M. truncatula*, which were isolated, stained and analyzed simultaneously. The *M. truncatula* genome (2n=2x=~860 Mb) served as an internal reference standard. The ratio of peak means was equal 3.49, hence the estimated genome size of autotetraploid cultivated alfalfa was 2n=4x=~3,001 Mb. The variation is usually expressed as the coefficient of variation (CV), and CVs below 5 are considered acceptable.

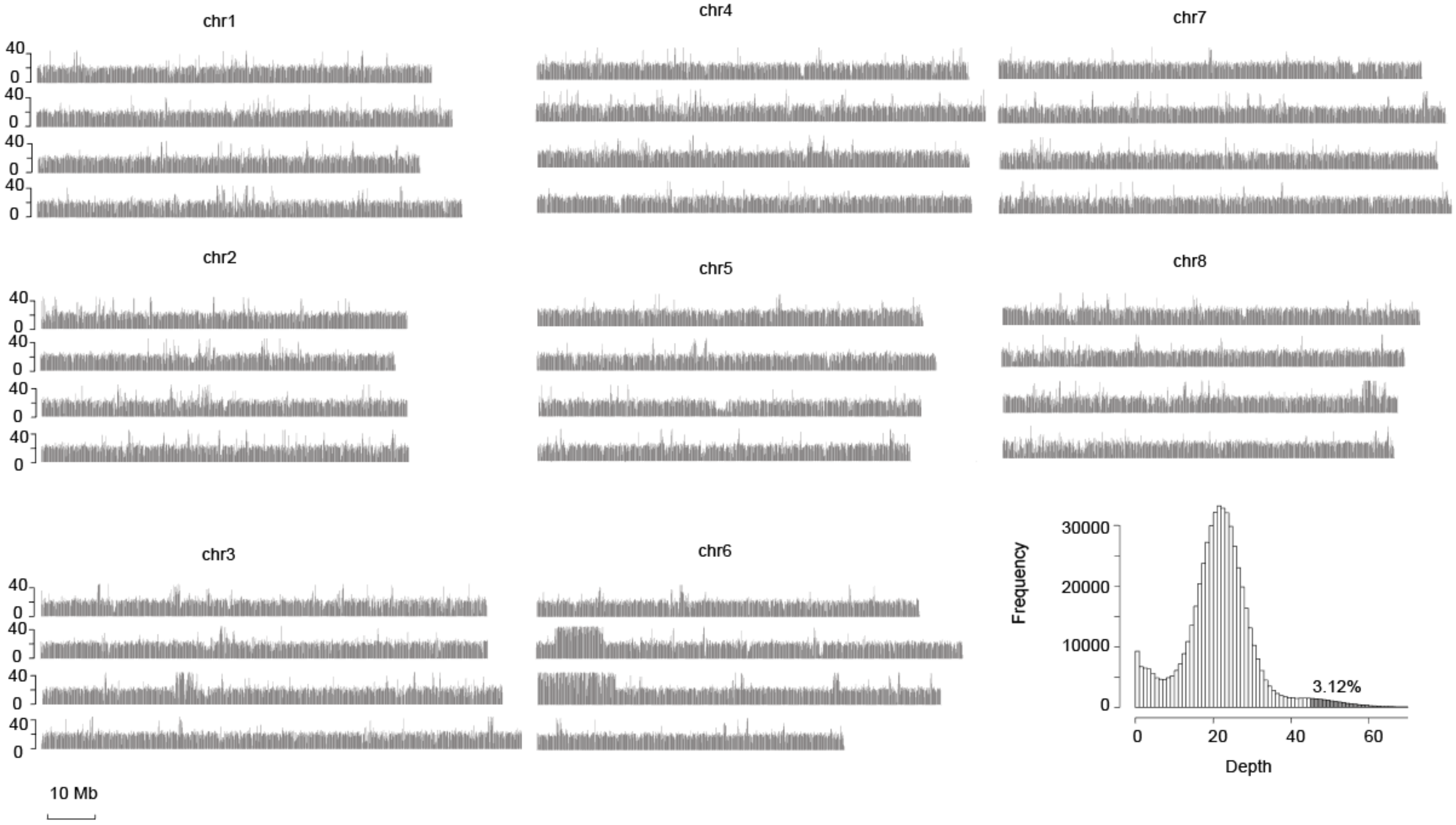

chr1
chr2
chr3
chr4
chr5
chr6
chr7
chr8
40
0
10 Mb
Frequency
30000
20000
10000
0
3.12%
0
20
40
60
Depth

Supplementary Figure 3. Coverage depth of assembled cultivated alfalfa genome. Chromosomes were binned into 5 kb window to display the average depth along assembled chromosomes. Each group consists of 4 chromosomes, and most windows have coverage depth ~22. The frequency-distribution of depth is shown in lower-right, only 3.12% windows have coverage depth >44 (shadowed bar).

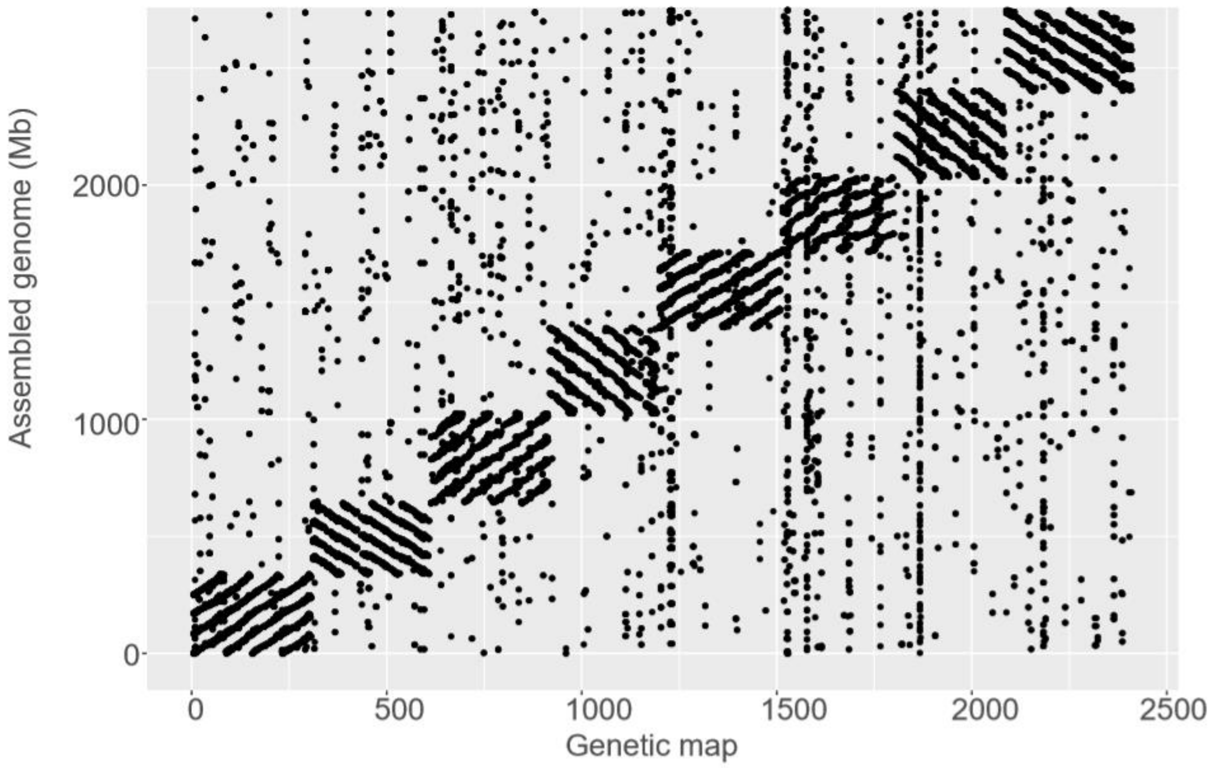

Assembled genome (Mb)
0
1000
2000
Genetic map
0
500
1000
1500
2000
2500

Supplementary Figure 4. Reference genetic map was mapped to assembled genome of cultivated alfalfa. Genetic map consists of 3,555 tag sequences were mapped to assembled chromosomes using blastn. Hits were filtered with e-value cutoff 1e-20. Although this genetic map is constructed using strains different from ours, 3,398 (95.6%) tag sequences can be mapped to our genome, each tag sequence has 9.8 hits on average. The intensive diagonal hit-blocks indicate this assembly is substantially concordant with the reference genetic map.

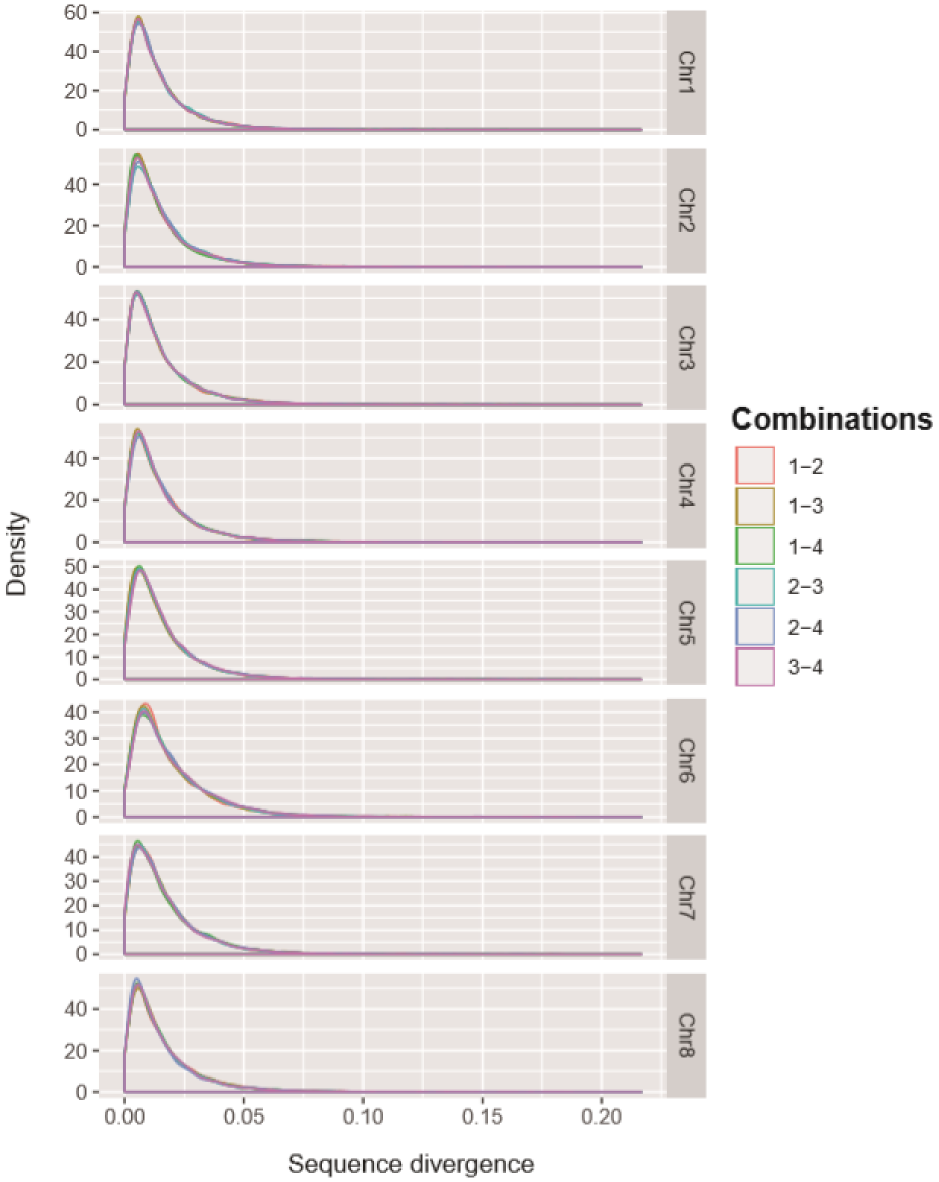

Supplementary Figure 5. Sequence divergence between allelic chromosomes. Each allelic chromosome was aligned to others using last with default parameters, sequence divergence was calculated for each alignment blocks and summarized. The distribution of sequence divergence peaks at ~0.01.

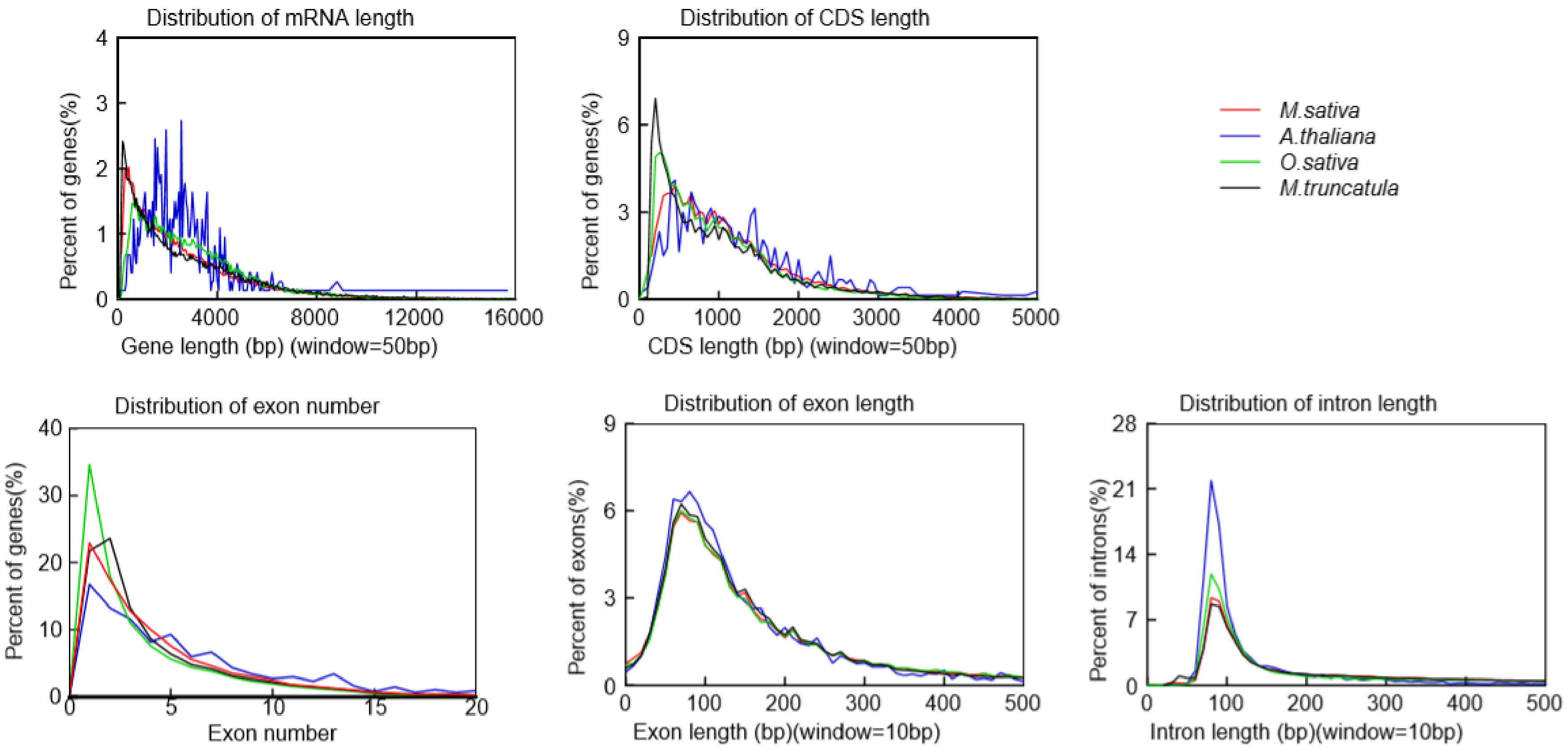

Supplementary Figure 6. Comparison of gene structure characters of cultivated alfalfa with three other species. Distributions of mRNA length, CDS lengths, exon numbers, exon lengths and intron lengths, all showing that *M. sativa* is highly similar to the three other species, which have been well-annotated.

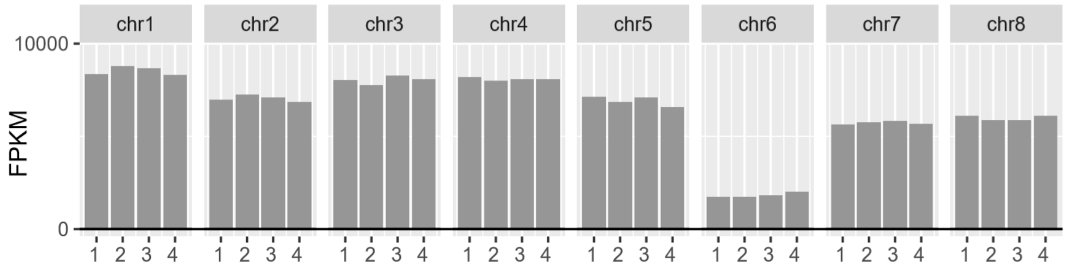

Supplementary Figure 7. Expression levels of genes with 4 alleles. Expression level was calculated in fragments per kilobase of exon model per million reads mapped (FPKM values).

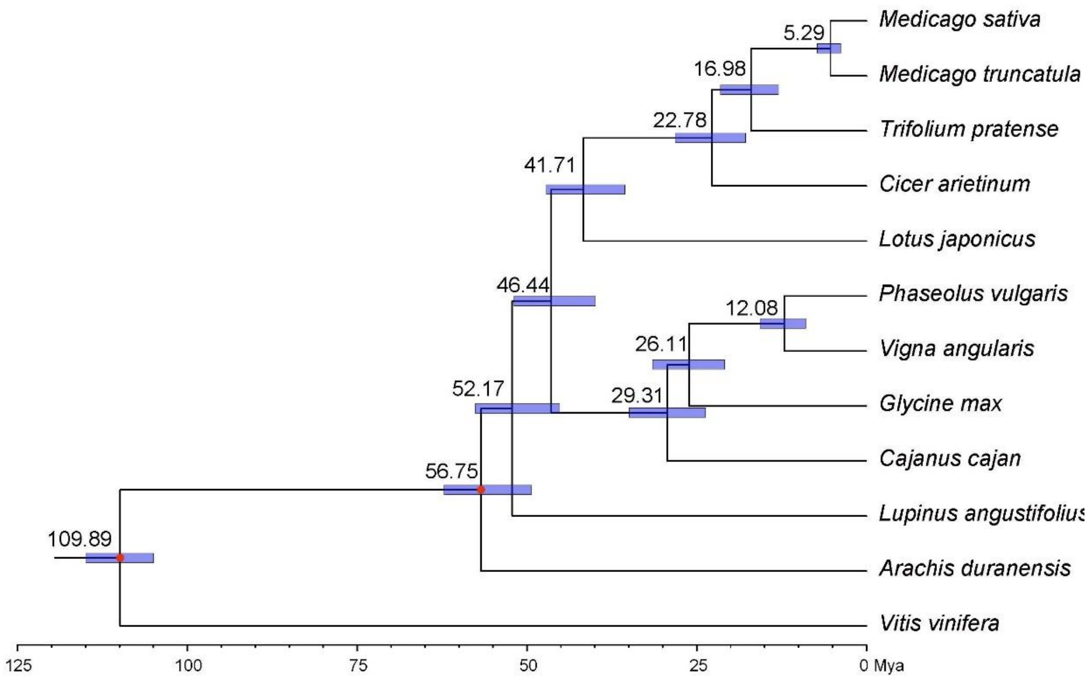

Supplementary Figure 8. Times of divergence of *Medicago sativa* and 11 other species. The 95% HPD split time is shown above each node and the red circles represent fossil calibrations (105~115 Mya for the *Vitis vinifera*-leguminous split and 49~62 Mya for the *Arachis duranensis*-other leguminous species split).

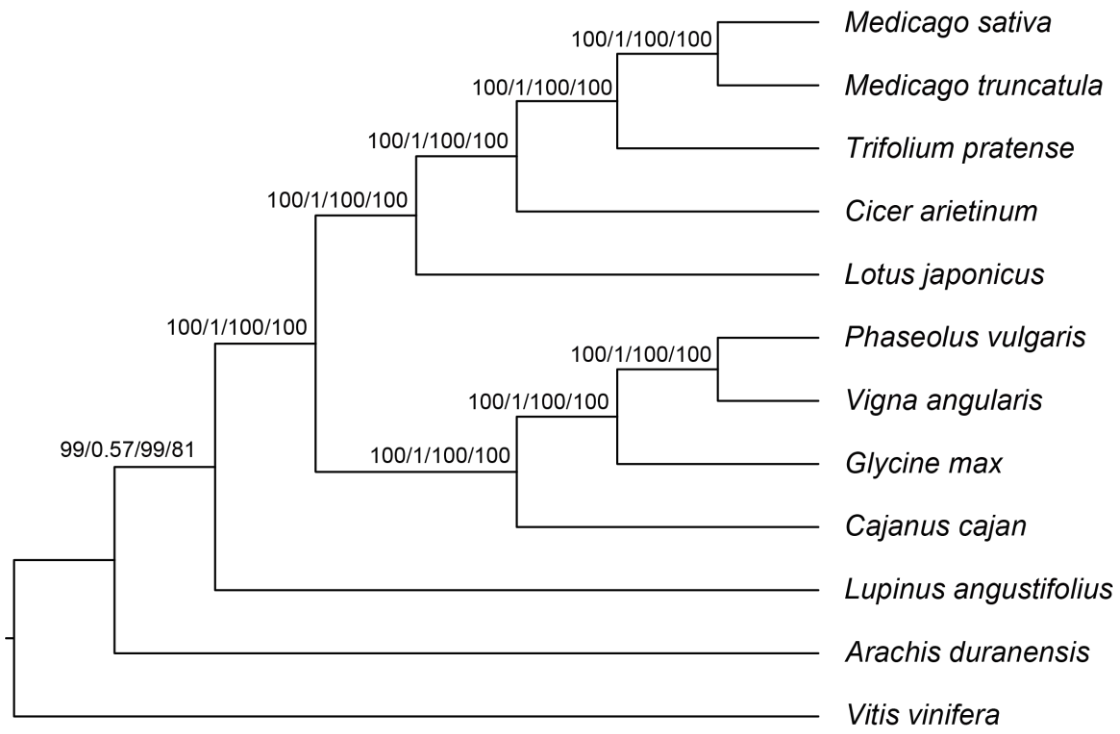

Supplementary Figure 9. Phylogenetic tree of *Medicago sativa* and the 11 other considered species. Bootstrap values and posterior probabilities are indicated for each internal branch, from left to right: OrthoMCL identified single copy genes with concatenation analysis using maximum likelihood (OSCGs-CA-ML), OSCGs with ASTRAL, low copy genes with STAG and BUSCO-identified conserved single copy genes with CA-ML.

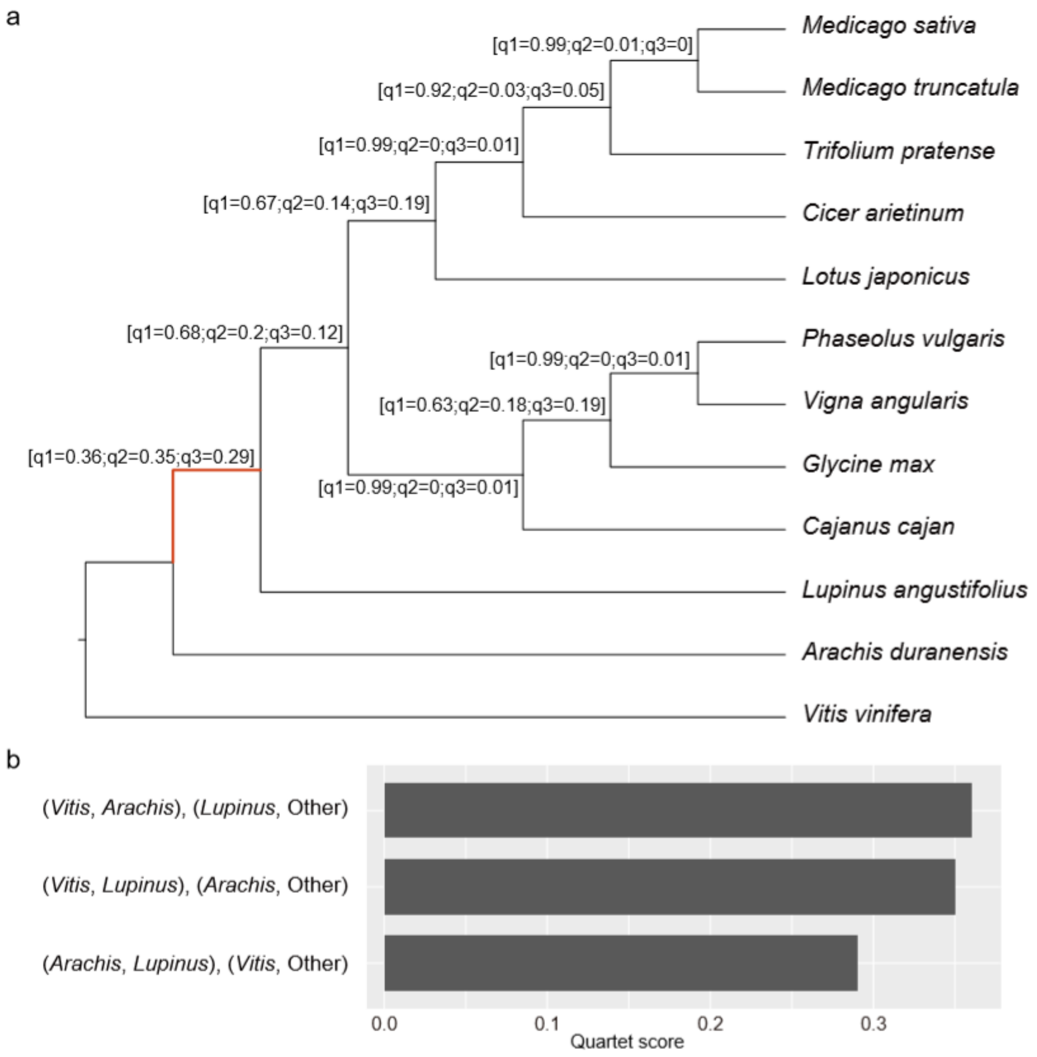

Supplementary Figure 10. Results of ASTRAL quartet-score analysis for all branches. a, Quartet scores were calculated for the three possible arrangements (q1 to q3) and are displayed above each branch. b, Quartet scores of the three possible topologies within the basal lineages *Arachis* and *Lupinus* (the red branch in a).

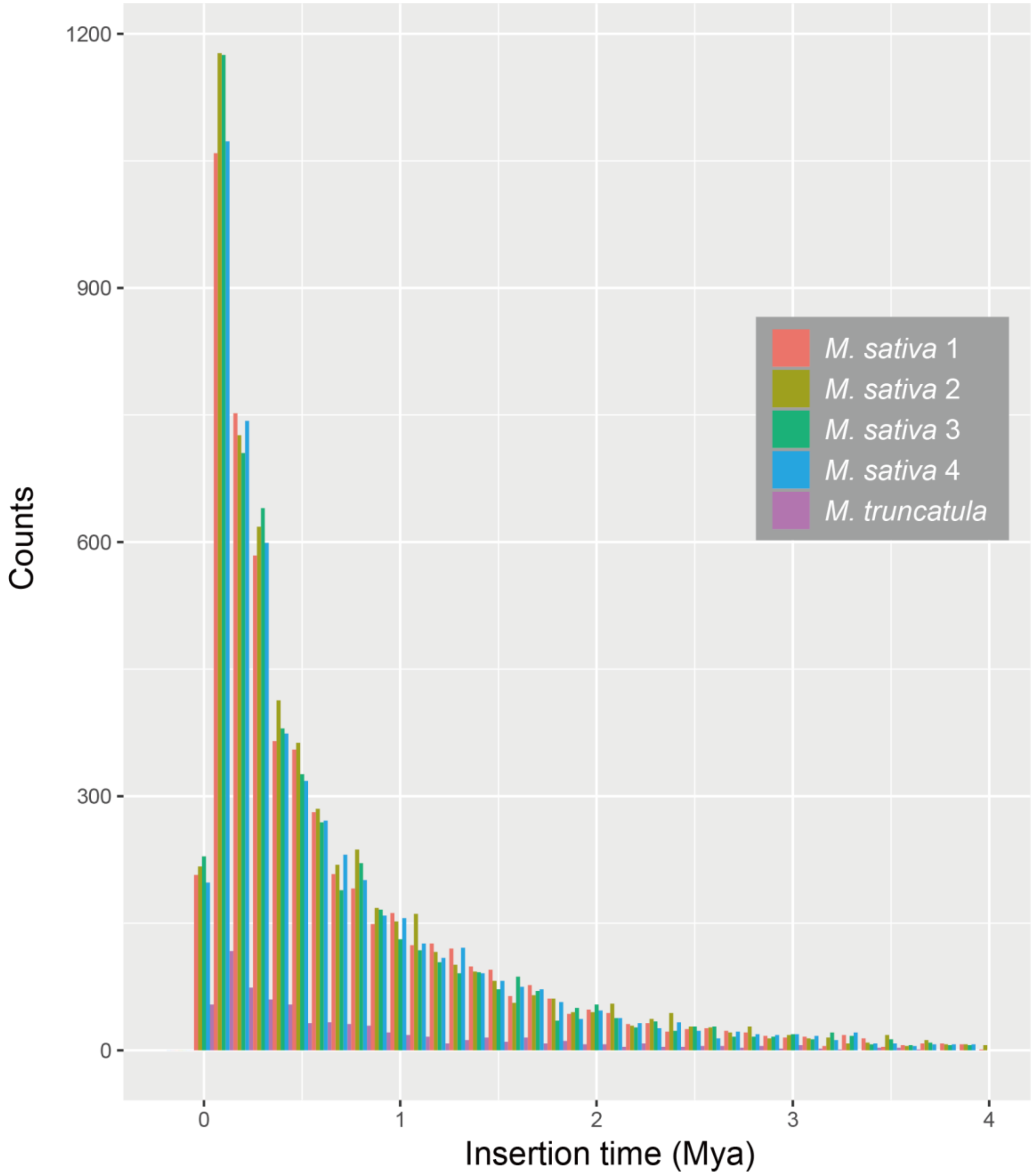

Supplementary Figure 11. Comparison of timing of LTR-RT insertions between each allelic alfalfa genome and *M. truncatula* genome. The cultivated alfalfa genome was split into four groups, each contains 8 chromosomes.

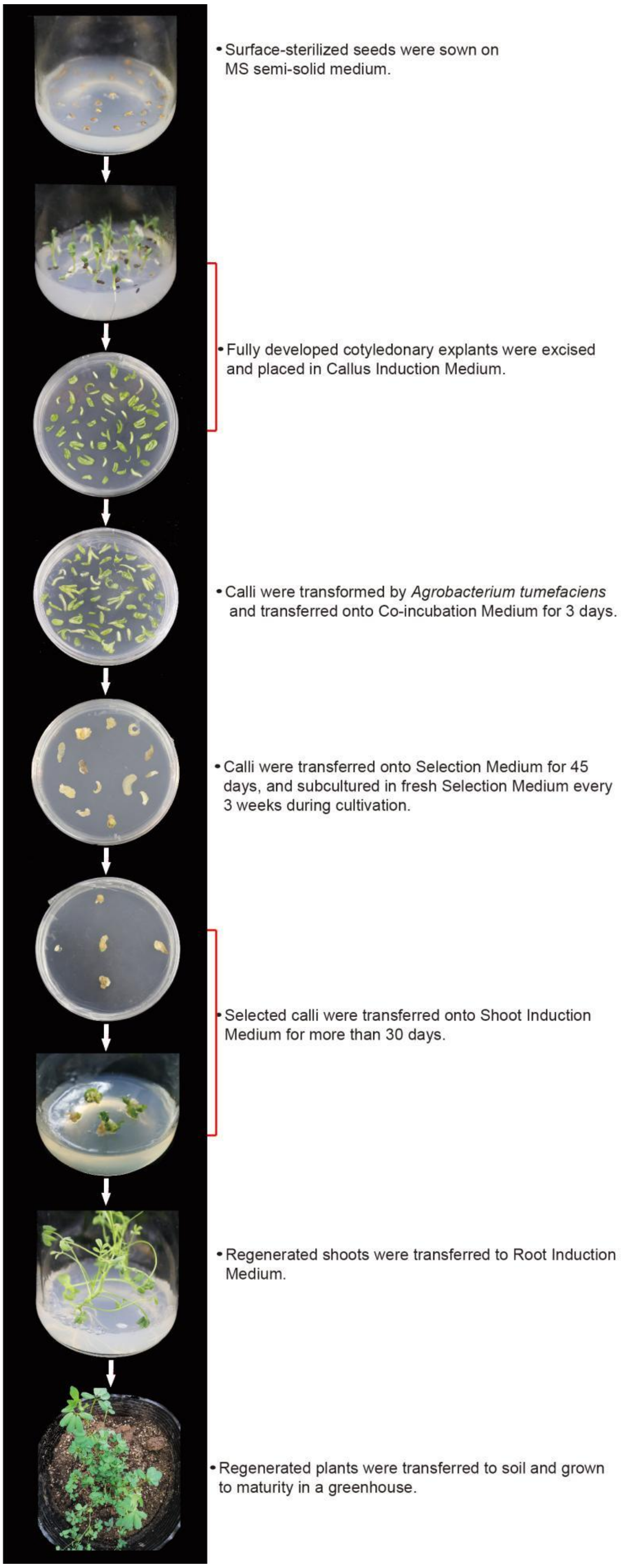

Supplementary Figure 12. Pipeline for transforming alfalfa by *Agrobacterium tumefaciens*.

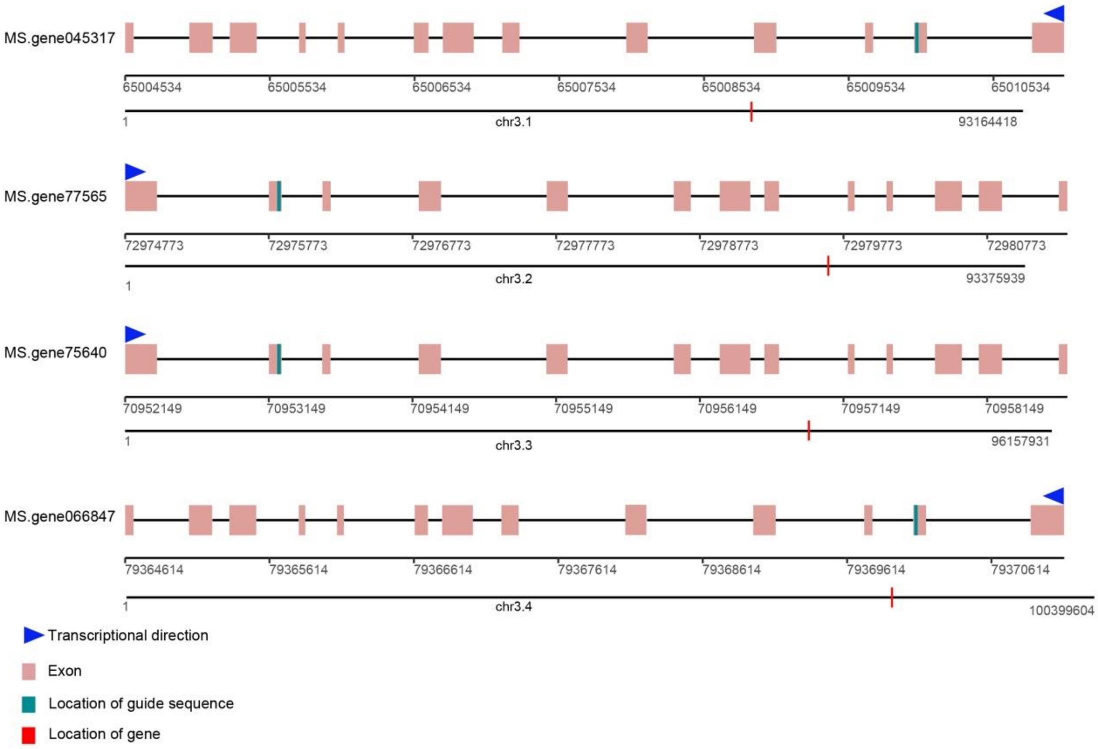

Supplementary Figure 13. Genomic positions of *MsPDS* alleles in the cultivated alfalfa genome.

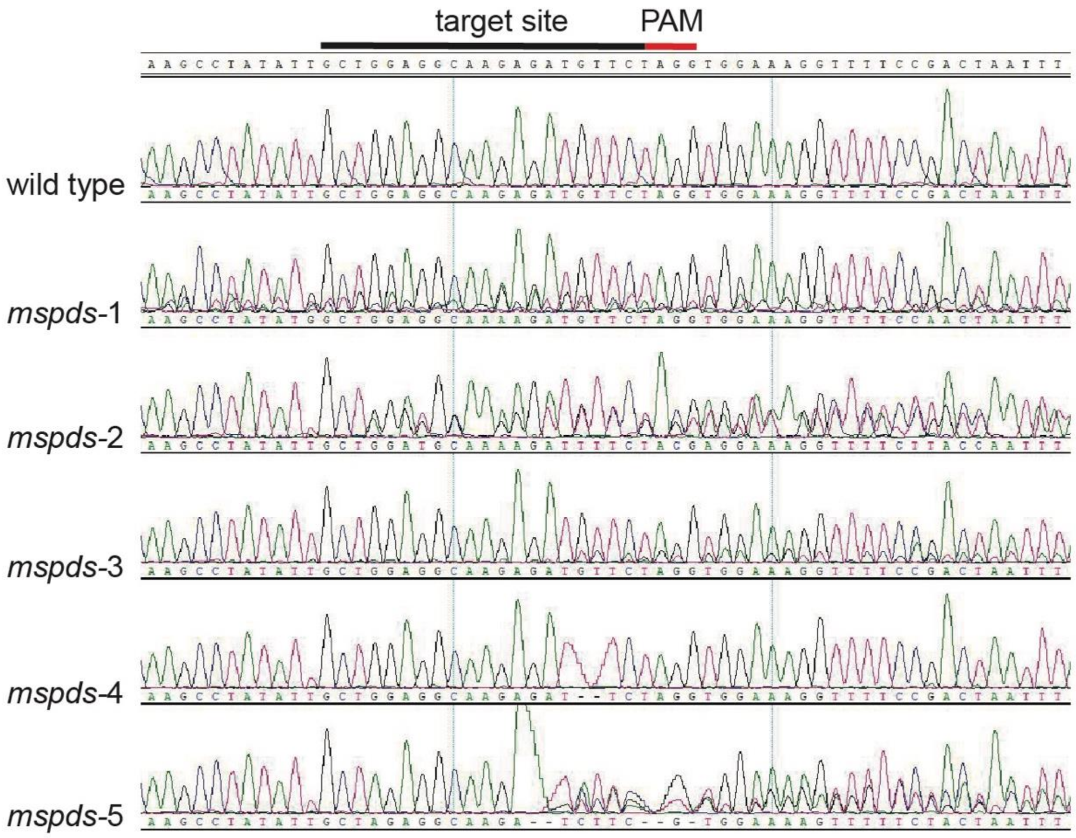

Supplementary Figure 14. Sequencing chromatograms of five candidate *mspds* mutants obtained by directly sequencing their PCR product.

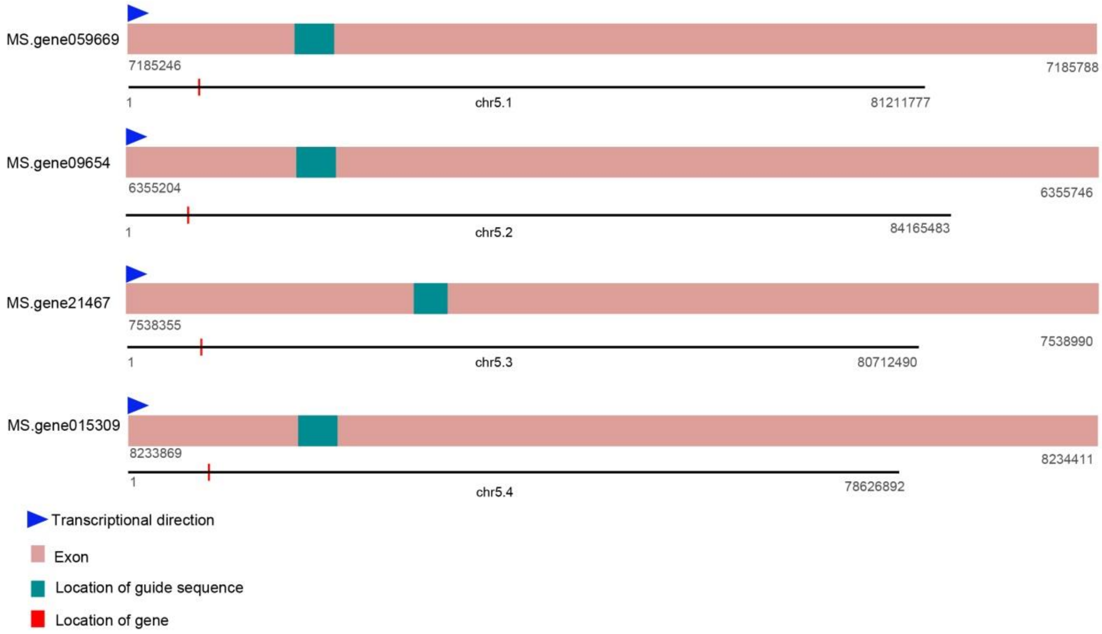

Supplementary Figure 15. Genomic positions of *MsPALM1* alleles in the cultivated alfalfa genome.

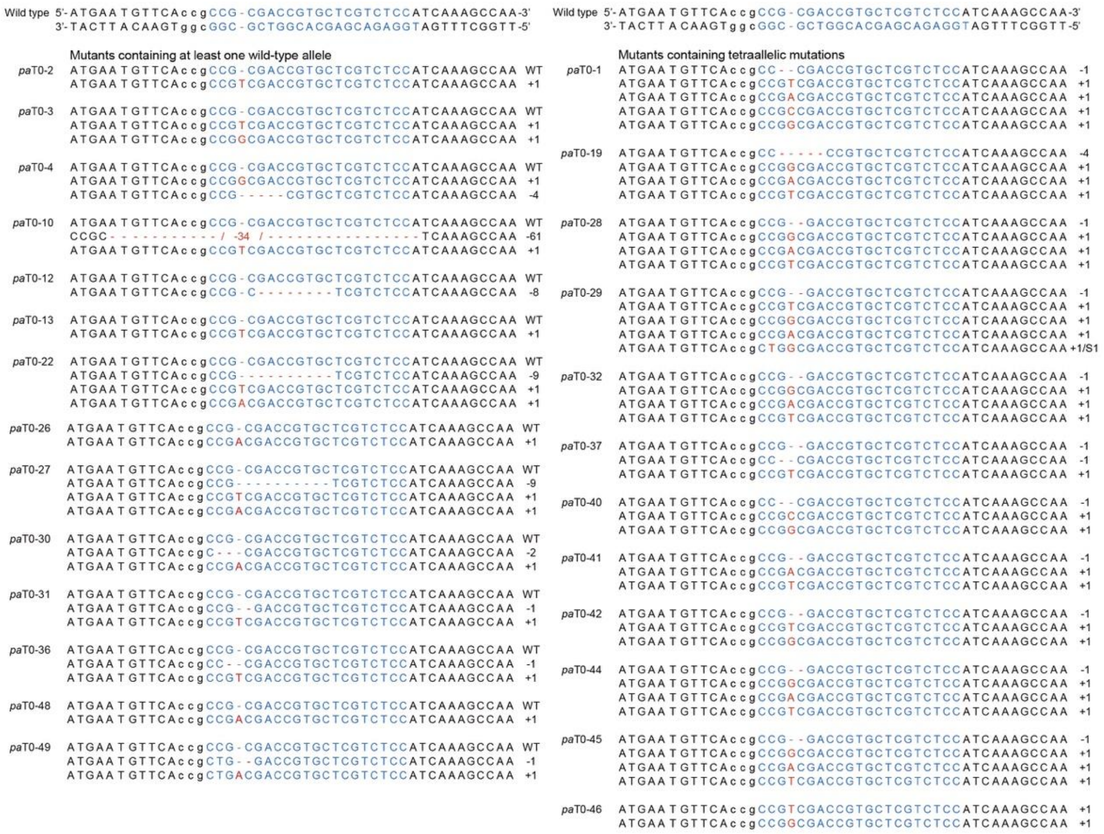

Supplementary Figure 16. Genotypes of all T0 *MsPALM1* mutants confirmed the presence of mutations at the target sites. Target sites are in blue text. The PAM regions are highlighted in black lowercase. Nucleotide deletions, insertions or substitutions are shown in red, with details to the right.

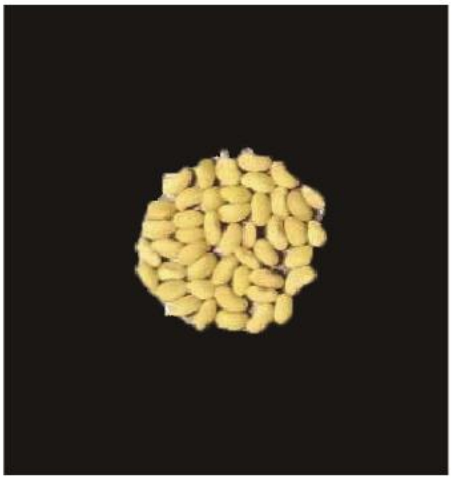

Supplementary Figure 17. Partial T1 seeds obtained by crossing two *palm1*-type mutants, *pa*T0-19 and *pa*T0-46.

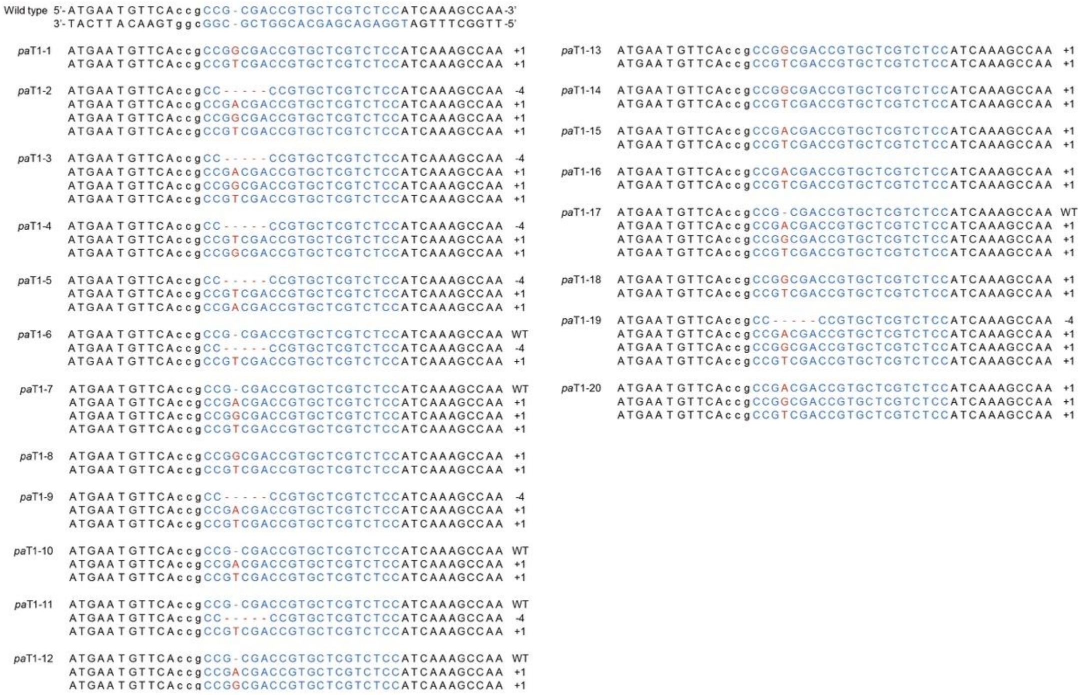

Supplementary Figure 18. Genotypes of *MsPALM1* T1 progenies confirmed the presence of parental mutations at the target sites. Target sites are in blue text. The PAM regions are highlighted in black lowercase. Nucleotide deletions, insertions or substitutions are shown in red, with details to the right.

Supplementary Table 1. Summary data of whole genome Illumina sequencing reads.

Library	Insert size	Raw reads		Qualified reads		SRA accession
		Number	Base (Gb)	Number	Base (Gb)	
Lib1	300	278,445,804	40.37	275,933,508	38.85	SRR9026572
Lib2	300	212,438,862	30.8	209,053,339	29.07	SRR9026571
Lib3	300	187,689,204	27.21	186,273,026	26.37	SRR9026574
Lib4	300	237,731,274	34.47	235,293,741	33.11	SRR9026573
Total	-	916,305,144	132.86	906,553,614	127.4	-

Supplementary Table 2. Summary data of PacBio CCS sequencing reads.

N10 (bp)	N50 (bp)	N90 (bp)	Total reads number	Total length (bp)	Accession code
15,104	12,604	11,125	5,542,606	70,400,851,464	SRR11285798

Supplementary Table 3. Summary data of Hi-C sequencing reads.

Accession code	Reads pairs	Read length (bp)	Total bases (Gb)
SRR9026577	743,892,194	150	223
SRR9026578	534,004,225	150	160

Supplementary Table 4. Unitigs of cultivated alfalfa which are syntenic to the *M. truncatula* genome were grouped for further clustering.

Chromosome of *M. truncatula*	Syntenic unitig number	Syntenic unitig length (bp)
chr1	601	259,740,699
chr2	491	219,178,684
chr3	585	272,931,013
chr4	681	270,443,606
chr5	471	234,324,977
chr6	225	162,071,019
chr7	537	251,116,830
chr8	495	214,466,120
Total	4,086	1,884,272,948

Supplementary Tables 5. Statistics of scaffolds after the first round of tuning.

Chr name of *M. truncatula*	Group number	Unitig length/number in each fine-tuned group
Chr1	4	57,553,913/115; 60,531,837/134; 67,767,116/154; 55,099,191/103
Chr2	4	46,637,283/81; 47,706,361/105; 47,058,605/94; 50,486,910/107
Chr3	4	61,822,163/115; 60,714,247/131; 62,415,712/104; 64,187,193/121
Chr4	8	49,864,266/99; 51,787,725/102; 45,279,189/102; 40,926,627/77; 18,832,641/51; 14,460,237/47; 20,151,663/61; 14,413,974/58
Chr5	4	57,413,145/107; 56,235,579/98; 53,840,417/98; 51,493,400/102
Chr6	4	37,257,111/51; 34,437,237/40; 48,435,127/70; 41,941,544/64
Chr7	4	53,784,163/98; 61,111,874/109; 63,081,455/144; 64,874,062/149
Chr8	8	36,460,828/75; 37,995,550/66; 39,110,064/77; 40,506,641/83; 12,270,565/34; 11,369,572/40; 13,155,195/29; 10,153,133/29
Total	40	1,765,914,955/3,540

Supplementary Table 6. Statistics of final chromosome-level scaffolds.

Chromosome name	Total length (bp)	Contig number
chr1.1	82,459,472	544
chr1.2	86,910,131	685
chr1.3	79,881,340	557
chr1.4	88,815,615	897
chr2.1	76,462,061	622
chr2.2	74,215,936	552
chr2.3	76,375,162	737
chr2.4	76,750,018	693
chr3.1	93,164,418	773
chr3.2	93,375,939	572
chr3.3	96,157,931	750
chr3.4	100,399,604	821
chr4.1	90,245,664	731
chr4.2	93,947,428	1,133
chr4.3	90,228,617	876
chr4.4	90,896,203	792
chr5.1	81,211,777	624
chr5.2	84,165,483	691
chr5.3	80,712,490	628
chr5.4	78,626,892	569
chr6.1	80,303,593	391
chr6.2	89,579,199	475
chr6.3	84,649,260	381
chr6.4	64,534,737	241
chr7.1	88,407,277	546
chr7.2	93,528,358	690
chr7.3	91,580,142	918
chr7.4	94,657,719	841
chr8.1	87,242,343	836
chr8.2	84,274,390	749
chr8.3	82,440,740	806
chr8.4	81,801,543	862

Supplementary Table 7. Statistics of sequenced Nanopore reads.

Library	N50 (bp)	N90 (bp)	Average (bp)	Total (Gb)	SRA accession no.
batch1	19,477	9,082	14,553	50.76	SRR9026576
batch2	23,896	11,373	17,982	48.72	SRR9026575
Total	21,577	9,922	16,052	99.49	-

Supplementary Table 8. Summary of BUSCOs recovered in the cultivated alfalfa genome.

BUSCO		All	sub-genome			
			1	2	3	4
Complete		97.16%	88.50%	88.30%	87.50%	87.20%
	Single	7.05%	80.90%	81.10%	79.60%	79.60%
	Duplicated	90.11%	7.60%	7.20%	7.90%	7.60%
Fragmented		0.22%	0.90%	0.80%	1.50%	1.30%
Missing		2.62%	10.60%	10.90%	11.00%	11.50%

Supplementary Table 9. Summary of RNA-seq and the mapping rate of *de novo* assembled transcripts.

Lib name (accession code)	Length cutoff	Total number	Aligned rate > 0.6		Aligned rate > 0.8		Aligned rate > 0.9	
			Transcript number	Percent	Transcript number	Percent	Transcript number	Percent
shoot-root (SRR9026570)	>200 bp	290,192	209,324	72.13%	205,944	70.97%	201,114	69.30%
	>500 bp	122,533	100,880	82.33%	99,027	80.82%	96,319	78.61%
	>1000 bp	58,874	53,026	90.07%	51,834	88.04%	50,438	85.67%
Leaf1 (SRR9026567)	>200 bp	135,573	132,008	97.37%	128,152	94.53%	123,654	91.21%
	> 500 bp	68,285	66,757	97.76%	64,756	94.83%	62,281	91.21%
	>1000 bp	36,843	36,178	98.20%	35,119	95.32%	33,826	91.81%
Leaf2 (SRR9026566)	>200 bp	143,711	139,352	96.97%	134,922	93.88%	130,154	90.57%
	>500 bp	69,909	68,102	97.42%	65,880	94.24%	63,292	90.53%
	>1000 bp	35,570	34,777	97.77%	33,641	94.58%	32,411	91.12%
Leaf3 (SRR9026569)	>200 bp	159,316	154,176	96.77%	149,466	93.82%	144,368	90.62%
	>500 bp	76,251	74,326	97.48%	72,002	94.43%	69,375	90.98%
	>1000 bp	39,519	38,710	97.95%	37,489	94.86%	36,245	91.72%
Leaf4 (SRR9026568)	>200 bp	148,438	144,023	97.03%	139,276	93.83%	134,257	90.45%
	>500 bp	72,455	70,642	97.50%	68,283	94.24%	65,634	90.59%
	>1000bp	37,823	37,026	97.89%	35,784	94.61%	34,582	91.43%

Supplementary Table 10. Comparison of gene structure characters between cultivated alfalfa and *M. truncatula*.

Species	Gene number	Average mRNA length (bp)	Average CDS length (bp)	Average exon number	Average exon length (bp)	Average intron length (bp)
Cultivated alfalfa	164,632	2,868.02	1,170.67	4.74	246.87	453.59
M. truncatula	57,585	2,890.82	1,038.34	4.48	231.94	437.45

Supplementary Table 11. Functional annotation of the predicted genes.

	Database	Number	Percent (%)
Total		164,634	100
Annotated	NR	163,810	99.50
	GO	91,338	55.48
	InterProscan	129,690	78.77
	Swissprot	115,789	70.33
	TrEMBL	162,963	98.99
	KEGG	88,116	53.52
Unannotated		751	0.46

Supplementary Table 12. Genomic content of *M. truncatula* and cultivated alfalfa and the contribution of each type of element to the difference in genome size.

Type		M. truncatula		Cultivated alfalfa (monoploid)		Contribution to inflated genome
		Length (bp)	Percentage	Length (bp)	Percentage	
CDS:		49,953,822	12.81%	46,767,984	6.83%	-
Intronic:						
	Gypsy	1,171,961	0.30%	1,661,382	0.24%	0.17%
	Copia	2,031,740	0.52%	2,672,970	0.39%	0.22%
	Non-LTR retro TE	2,013,460	0.52%	2,091,445	0.31%	0.03%
	DNA TE	5,460,050	1.40%	5,450,279	0.80%	-
	Unknown TE	5,864,904	1.50%	3,337,022	0.49%	-
	Simple/Tandem repeat	2,052,611	0.53%	1,993,321	0.29%	-
	Non-repeat	48,480,930	12.43%	48,931,930	7.15%	0.15%
Intergenic:						
	Gypsy	29,284,343	7.51%	123,335,421	18.02%	31.93%
	Copia	19,660,270	5.04%	59,586,891	8.71%	13.56%
	Other retro TE	16,906,009	4.34%	27,333,689	3.99%	3.54%
	DNA TE	29,938,777	7.68%	50,161,372	7.33%	6.87%
	Unknown TE	36,690,304	9.41%	33,521,007	4.90%	-
	Simple/Tandem repeat	11,469,419	2.94%	40,702,772	5.95%	9.93%
	Non-repeat	128,993,929	33.08%	207,249,509	30.28%	26.57%
Total:		389,972,529		684,500,171		

Note: The total assembled content/4 as an average content for each monoploid.

Supplementary Table 13. Summary of results of whole genome sequencing of three mutants.

Mutant	Number of reads	Number of Bases	Read length(bp)	Q20(%)*	Q30(%)**	GC content(%)***
*pa*T0-1	231,849,818	34,777,472,700	150	97.7%	93.3%	34.9%
*pa*T0-19	236,279,130	35,441,869,500	150	97.8%	93.5%	35.1%
*pa*T0-46	181,322,078	27,198,311,700	150	97.7%	93.3%	34.9%

*Q20 Percentage of bases with quality higher than 20

**Q30 Percentage of bases with quality higher than 30

***GC content: Percentage of G and C bases

Supplementary Table 14. Summary of variations detected in three sequenced mutants.

No. of mismatches in potential off-target sites	No. of off-target sites with SNPs or indels (No. of detected off-target sites containing ≥1 bp mismatch in seed sequence)*	Total No. of SNPs (No. of SNPs located in coding regions)			Total No. of indels (No. of indels located in coding regions)		
		*pa*T0-1	*pa*T0-19	*pa*T0-46	*pa*T0-1	*pa*T0-19	*pa*T0-46
1	0(0)	0(0)	0(0)	0(0)	0(0)	0(0)	0(0)
2	0(0)	0(0)	0(0)	0(0)	0(0)	0(0)	0(0)
3	0(0)	0(0)	0(0)	0(0)	0(0)	0(0)	0(0)
4	11(11)	6(3)	5(4)	5(1)	0(0)	0(0)	0(0)
5	67(67)	30(5)	33(11)	28(5)	1(0)	0(0)	1(0)

* Two or three sequenced mutants share common variations in some common off-target sites.

Supplementary Table 15. Primers used in this study.

Primer name	Primer sequence(5'-3')	Application
MsPD-F	ATCCAACAATGAGTGAAGGCTTTAT	Amplifying the *MsPDS* target site
MsPD-R	AGTCTCCATCTTCATCTTTCCAT	
MsPA-F	AATTTTCATCCCCCACCCCCATTA	Amplifying the *MsPALM1* target site
MsPA-R	TTCTCTACACACTGAAAAAGAGAGA	
Ca-F	CTCCGCCGTCAATGTAGCC	Amplifying the *hSpCas9* for detecting the exit of T-DNA in progenies.
Ca-R	GCCCACATGATCAAGTTCCG	
Hp-F	TTCCGGAAGTGCTTGACATTGGGGA	Amplifying the *Hpt* for detecting the exit of T-DNA in progenies.
Hp-R	ACGGTGTCGTCCATCACAGTTTGCC	
Vt-F	GGCATGCAAGCTTATCGATAC	Detecting the successful ligation of targets into pMs-CRISPR/Cas9
