# Supplementary material for: Allele-aware chromosome-level genome assembly and efficient transgene-free genome editing for the autotetraploid cultivated alfalfa
Source: Nat Commun. 2020 May 19;11:2494. doi: 10.1038/s41467-020-16338-x (PMC7237683; doi:10.1038/s41467-020-16338-x)
Supplement: Supplementary file 3 — Reporting Summary [file 41467_2020_16338_MOESM3_ESM.pdf]

nature research

Corresponding author(s): Qiang Qiu, Wen Wang and Jianghua Chen.
NCOMMS-19-27438B

Last updated by author(s): Apr 19, 2020

Reporting Summary

Nature Research wishes to improve the reproducibility of the work that we publish. This form provides structure for consistency and transparency in reporting. For further information on Nature Research policies, see Authors & Referees and the Editorial Policy Checklist .

Statistics

For all statistical analyses, confirm that the following items are present in the figure legend, table legend, main text, or Methods section.

n/a	Confirmed	
☐	☒	The exact sample size (*n*) for each experimental group/condition, given as a discrete number and unit of measurement
☐	☒	A statement on whether measurements were taken from distinct samples or whether the same sample was measured repeatedly
☐	☒	The statistical test(s) used AND whether they are one- or two-sided<br>*Only common tests should be described solely by name; describe more complex techniques in the Methods section.*
☒	☐	A description of all covariates tested
☐	☒	A description of any assumptions or corrections, such as tests of normality and adjustment for multiple comparisons
☐	☒	A full description of the statistical parameters including central tendency (e.g. means) or other basic estimates (e.g. regression coefficient) AND variation (e.g. standard deviation) or associated estimates of uncertainty (e.g. confidence intervals)
☒	☐	For null hypothesis testing, the test statistic (e.g. *F*, *t*, *r*) with confidence intervals, effect sizes, degrees of freedom and *P* value noted<br>*Give P values as exact values whenever suitable.*
☐	☒	For Bayesian analysis, information on the choice of priors and Markov chain Monte Carlo settings
☐	☒	For hierarchical and complex designs, identification of the appropriate level for tests and full reporting of outcomes
☒	☐	Estimates of effect sizes (e.g. Cohen's *d*, Pearson's *r*), indicating how they were calculated

Our web collection on statistics for biologists contains articles on many of the points above.

Software and code

Policy information about availability of computer code

Data collection	No software was used for data collection.
Data analysis	Canu software package; ALLHiC; Trimmomatic (v. 0.36); Kmerfreq in the SOAPec (v. 2.01) package; HiC-Pro; MCscan in Jcvi; Juicebox; TRF (v. 4.07b); RepeatProteinMask; RepeatMasker (v. 4.0.5); LTR_FINDER; Blast (v. 2.2.26); GeneWise in the Wise2 package (v. 2.2.0); Blat (v. 34); GMAP (v. 2016-11-07); PASA (v. 2.2.0); TransDecoder; AUGUSTUS (v. 3.2.2); GeneID (v. 1.4.4); GlimmerHMM (v. 3.0.2); SNAP (v. 2006-07-28); EVM (v. 2012-06-25); InterProScan (v. 5.17-56.0); MCScanX; OrthoMCL; MUSCLE; RAxML; ASTRAL; STAG; PAML mcmctree program; Tracer v. 1.4 (http://beast.community/tracer); home-made scripts (https://github.com/stanleyouth/-/blob/master/crispr.sgRNA.finder.pl); sgRNAcas9 (v. 3.0.5); FastQC (https://www.bioinformatics.babraham.ac.uk/projects/fastqc/); Bwa (v. 0.7.12); Picard tools (v1.119, https://broadinstitute.github.io/picard/); GATK v. 3.5; SnpEff; SnapGene; FlowJo.v7.6.3; Mega 6; Oligo 7.37; DNASTAR package; EndNote X7; Adobe Illustrator CS5; Microsoft Excel 2010

For manuscripts utilizing custom algorithms or software that are central to the research but not yet described in published literature, software must be made available to editors/reviewers. We strongly encourage code deposition in a community repository (e.g. GitHub). See the Nature Research guidelines for submitting code & software for further information.

Data

Policy information about availability of data

All manuscripts must include a data availability statement. This statement should provide the following information, where applicable:

- Accession codes, unique identifiers, or web links for publicly available datasets
- A list of figures that have associated raw data
- A description of any restrictions on data availability

All genome and transcriptome sequencing raw data described in this article are publicly available in the NCBI database under project PRJNA540215 [https://www.ncbi.nlm.nih.gov/bioproject/PRJNA540215], and the genome assembly files are available at https://figshare.com/projects/whole_genome_sequencing_and_assembly_of_Medicago_sativa/66380. Data supporting the findings of this work are available within the paper and its Supplementary Information files. A reporting summary for this article is available as a Supplementary Information file. The source data underlying Figs. 1, 4c, f and h

are provided as a Source Data file.

Field-specific reporting

Please select the one below that is the best fit for your research. If you are not sure, read the appropriate sections before making your selection.

☒ Life sciences ☐ Behavioural & social sciences ☐ Ecological, evolutionary & environmental sciences

For a reference copy of the document with all sections, see nature.com/documents/nr-reporting-summary-flat.pdf

Life sciences study design

All studies must disclose on these points even when the disclosure is negative.

Sample size	The size of samples used for sequencing depends on the sequencing libraries. And samples used for transformation were fully developed cotyledonary explants from 7-days-old seedlings, since the fully developed cotyledons from 7 to 14-days-old seedlings are the best materials for inducing calli used for transformation.
Data exclusions	No data was excluded.
Replication	Various approaches are used to evaluate the genome assembly of cultivated alfalfa, confirming the assembly is of high-quality. To test the repeatability of the established CRISPR/Cas9 protocol, two endogenous genes (MsPDS and MsPALM1) are chosen as replicated targets, and 5 and 26 mutants are created for them respectively. The results of these two genes illustrate the repeatability of this established CRISPR/Cas9 protocol. To evaluate the off-target effects, three palm1-type mutants were resequenced and analyzed by globally scanning of their whole genomes. No off-target mutations in protein-coding regions are detected in these mutants, which illustrates the specificity of our protocol.
Randomization	Samples were chosen randomly.
Blinding	Blinding was not relevant to our study since our study is on genome and transcriptome analysis and genome editing of plant species.

Reporting for specific materials, systems and methods

We require information from authors about some types of materials, experimental systems and methods used in many studies. Here, indicate whether each material, system or method listed is relevant to your study. If you are not sure if a list item applies to your research, read the appropriate section before selecting a response.

Materials & experimental systems

n/a	Involved in the study	
☒	☐	Antibodies
☒	☐	Eukaryotic cell lines
☒	☐	Palaeontology
☒	☐	Animals and other organisms
☒	☐	Human research participants
☒	☐	Clinical data

Methods

n/a	Involved in the study	
☒	☐	ChIP-seq
☐	☒	Flow cytometry
☒	☐	MRI-based neuroimaging

Flow Cytometry

Plots

Confirm that:

☒ The axis labels state the marker and fluorochrome used (e.g. CD4-FITC).

☒ The axis scales are clearly visible. Include numbers along axes only for bottom left plot of group (a 'group' is an analysis of identical markers).

☒ All plots are contour plots with outliers or pseudocolor plots.

☒ A numerical value for number of cells or percentage (with statistics) is provided.

Methodology

Sample preparation	Leaves from M. sativa (cultivar XinJiangDaYe) and M. truncatula (cultivar Jemalong, A17) plants were finely chopped together with a razor blade in 400 µl Galbraith buffer with 5 µl•mL-1 β-mercaptoethanol. The resulting suspension was filtered through 30-µm nylon. From a 500 U/mL stock of Ribonuclease A, 10 µl•mL-1 was added, and propidium iodide was added to 50 µg•mL-1. After 30 min incubation at room temperature, the DNA peak ratio was assessed by flow cytometry.

Instrument	LSR Fortessa flow cytometer, Becton Dickinson Biosciences, USA
Software	FlowJo.v7.6.3
Cell population abundance	Histogram of relative fluorescence intensities of PI-stained nuclei of M. sativa and M. truncatula is used to evaluate the genome size of M. sativa. The means of fluorescence intensities of PI-stained nuclei of M. truncatula and M. sativa are 206 and 718, respectively. The variation is usually expressed as the coefficient of variation (CV), and CVs below 5 are considered acceptable. CVs of M. truncatula and M. satuiva are 3.06 and 2.09, respectively.
Gating strategy	FSC-A,SSC-A subset/FSC-A, PE-A subset/PE-W, PE-A subset/PE-A, Histogram

☒ Tick this box to confirm that a figure exemplifying the gating strategy is provided in the Supplementary Information.
