# Supplementary material for: Allele-aware chromosome-level genome assembly and efficient transgene-free genome editing for the autotetraploid cultivated alfalfa
Source: Nat Commun. 2020 May 19;11:2494. doi: 10.1038/s41467-020-16338-x (PMC7237683; doi:10.1038/s41467-020-16338-x)
Supplement: Supplementary file 4 — Description of Additional Supplementary Files [file 41467_2020_16338_MOESM4_ESM.docx]

**Description of Additional Supplementary Files**

File Name: Supplementary Data 1

Description: Statistics of Hi-C reads connection map. The inter- and intra-chromosomal connection numbers for each chromosome are calculated and listed.

File Name: Supplementary Data 2

Description: Genomic content of each assembled allelic chromosome of cultivated alfalfa, showing that each allelic chromosome has similar genomic content.

File Name: Supplementary Data 3

Description: Guide sequences designed for *MsPDS* using home-made scripts. The selected guide sequence is marked with a red asterisk.

File Name: Supplementary Data 4

Description: Guide sequences designed for *MsPALM1* using home-made scripts. The selected guide sequence is marked with a red asterisk.
