# Supplementary material for: Allele-aware chromosome-level genome assembly and efficient transgene-free genome editing for the autotetraploid cultivated alfalfa
Source: Nat Commun. 2020 May 19;11:2494. doi: 10.1038/s41467-020-16338-x (PMC7237683; doi:10.1038/s41467-020-16338-x)
Supplement: Supplementary file 2 — Peer Review File [file 41467_2020_16338_MOESM2_ESM.pdf]

Reviewers' comments:

Reviewer #1 (Remarks to the Author):

The manuscript describes results of research to generate a reference genome for the tetraploid obligatory outcrossing plant alfalfa. The author(s) used a combination of single molecule sequencing, genetic maps and Hi-C data to build a reference genome that accounted for roughly 81.5% of the estimated alfalfa genome. They then used the assembled genome sequence to design sgRNA for two genes, PDS and PALM1, for use in CRISPR/Cas9 mutagenesis. With the PDS mutagenesis, they obtained mutants having a dwarf albino phenotype typical of pds null mutants. Further, molecular analysis revealed PDS alleles were indeed mutated in three transgenic plants . In the second CRISPR/Cas9 mutagenesis experiment, they targeted PALM1 gene which is known to control the valuable trait of leaf development (loss of function produces pentafoliate instead of trifoliate), and thus nutritional quality, as proteins accumulate predominantly in the leaves in alfalfa. The authors were able to obtain null palm1 mutants and showed their inheritance in the progeny.

While I do not feel fully qualified to assess the technical aspects of the compilation of the alfalfa reference genome, I recognize assembling this genome is as a major achievement given its large size and tertraploid nature. A number of transcript databases for alfalfa have been published over the years (e.g. Zhang et al, 2015. PLoS One 10:e0122170; O'Rourke et al, 2015, BMC Genomics16:502), but to my knowledge no full alfalfa genome sequence has been made public yet. Researchers have thus had to rely heavily on the genome of the related model plant Medicago truncatula. The publication of the alfalfa reference genome described in this study should therefore allow for major advances in alfalfa research.

Establishing efficient CRISPR/Cas9-based mutagenesis in alfalfa is a major advance, even though previous reports have shown the feasibility of CRISPR/Cas9 mutagenesis in this crop (Gao et al., 2018; Planta 247:1043), albeit with a lower mutagenesis efficiency than reported in this study. The authors followed well established procedures to select sgRNA, make the constructs, and generate/characterize the mutants. What is surprising (and encouraging) is the fact that they obtained null mutants in T0 that were genetically heritable and the pentafoliate phenotype of palm1mutants was stable in the progeny. It is noteworthy that some of the T1 palm1 mutants were transgene-free. This is sort of unexpected in an organism with a large tertraploid genome and obligatory outcrossing reproductive behaviour.

Although the manuscript is well written and is technically sound, I have some concerns regarding the following points:

1. Genome editing frequency: It would have been useful to have an idea of the genome editing frequency on a global scale. The authors chose 50 transgenic plants for PDS and 49 for PALM1, for analysis of genome editing using conventional PCR-sequencing technique. A more robust method such as Droplet Digital PCR would have been more informative of genome editing rates/efficiency (Mock et al., 2016, Nat Protoc 11:598).
2. Off-target effects/specificity: The authors assessed off-target effects by screening candidate sgRNA using sgRNACas9 software (i.e. in silico analysis). While this could be considered as a first step towards predicting the presence/absence of off target effects, it does not provide a conclusive proof. To determine off-targets of sgRNA a more comprehensive genome-wide analysis of the mutant by Next Generation Sequencing may be necessary. This is especially true since the authors are relying on a partial assembled genome.
3. While the figures are generally well illustrated and presented, I found it hard to follow Figure 3b; it is too crowded with small fonts. Perhaps some of the information can be presented as supplementary data.
4. The manuscript should undergo at least one more round of editing to correct minor grammar errors. An example would be the word "blasting" on line 443. BLAST is not a verb. The correct wording

should be “conducting a BLAST search”.

Reviewer #2 (Remarks to the Author):

The manuscript entitled, "Chromosome-level reference genome and efficient trangene-free genome editing of autotetraploid alfalfa (Medicago sativa L.0 using CRISPR/Cas9", describes a high quality assembly of a difficulty genetically complex species, deduction of genomic features relevant to the species origin and genome-size expansion, and demonstration of a methodology for CRISPR/Cas9 nucleotide modification.

In general, I am positively inclined towards publication of the document. With the exception of the occasional grammatical error, the document is written in a very approachable style and with appropriate sophistication for a high profile manuscript.

One question that occurred to me several times during my review was, would this manuscript not be better suited by a technology journal such as Nature Biotechnology? I think it is a relevant question, because the authors clearly have genome modification for crop improvement in mind, and despite the apparent high quality nature of the genome assembly this no longer constitutes novel biology which one would normally expect of a Nature Communications article. This comment aside, I provide a set of comments below that the authors might consider in revision.

1. In the Introduction on page 2 (lines 36-44), the authors are mistaken in the notion that Medicago truncautla was developed as a model for alfalfa. This was decidedly NOT one of the rationale. (Perhaps others have subsequently made this statement, but if so then they were mistaken). The sole rationale was to develop a tractable legume to study symbiosis. To the extent that Medicago truncatula was envisioned as a model for legumes, this came later and even then we thought of truncatula being a model for all legumes (not alfalfa in particular). It was only with the involvement of the Noble Foundation, and a few colleagues at USDA-ARS, that the idea of using truncatula as a model for sativa gained traction (and then it remained a small part of a larger rationale).

OK, sorry for the history lesson, but please revise the second paragraph in the discussion to remove statements about the rational for Medicago truncatula.

2. On page 4 (lines 72-72), the authors state that 89.1% of BUSCO homologs were identified. But it seems important to distinguish cases were all orthologs (4X) were encountered (which would be the actual rate of discovery), versus cases where assembly was adequate to capture only a subset (corrected for rates of actual loss).

3. Page 5, lines 86-87. Note that the chromosome 4 vs 8 translocation that the authors report is a genomic feature specifically of the Medicago genotype (A17) that was sequenced in the truncatula genome project. The base state of the truncatula species does not have this rearrangement. I think it is important to clarify this.

4. Page 8, line 116, and Figure 2d. It is not possible from Figure 2d for the reader to understand that certain classes of reverse transcriptases are expanded in the Ms subgenomes relative to Mt. I would suggest two corrections: 1. Improve the clarity of the figure, and 2. Provide a table in which the RTs are divided into clades and in each clade the frequency of Msa, Msb vs Mt homologs are enumerated – or some other quantitative presentation to back up the authors assertion about TE expansion.

Also, in the same paragraph, line 123, please provide the quantitative data that TE insertions are the main reason for genome expansion. What is the size difference of each subgenome relative to Mt and what percent of the size difference is attributable to TEs?

5. Page 8, lines 124 and on. I am a bit concerned about the phylogenetic analyses. Given that all legumes under comparison derive from an ancient polyploidization event, followed by diploidization, then the usefulness of singleton orthologs for phylogenetic analysis will be a function of the timing of loss relative to speciation (i.e., whether or not the retained singletons represent a coherent common history or not). I know from previous work that in some cases, return to a single copy state occurred after speciation, with different paralog histories in the different genomes.

Assuming that I am expressing my concern adequately, then my question, how can one derive a realistic phylogenetic analysis? I am struck by the accuracy of the resulting tree, which suggests to me that these genes became single copy before the species radiated. Is there evidence of this in the data? There are methods for looking at phylogenetic coherence between genes and I think that this is an important issue.

6. Page 7, line 132, the basal nature of Arachis and Lupinus is not novel, nor in question, and should not be presented as if this is a novel result. What you have learned is that your analysis recapitulates known relationships.

7. Page 8 lines 150-151 and line 155, page 10 lines 207-210, and page 13 lines 258-259. The notion that specific gene functions might be well enough understood or impactful enough to modulate nitrogen fixation, abiotic stress or nutrition is overly speculative. Certainly these can be described as traits that one might be interested in modifying, but there is no credible evidence that DNA repair (for example) might improve nutritional quality or that simply altering leaf morphology will have a significant impact on nutritional value. (I am aware of the Palmate mutants in Mt and I consider the role in plant nutrient content to be significantly oversold). Similarly, the suggestion that finding 2 copies of 22 sym genes (in Ms relative to Mt) might lead to insights in nitrogen fixation is very, very over simplistic.

If feel quite strongly that one needs to separate the proof that one can modify genetic targets with CRISPR, which the authors show with data, from the highly speculative (to the point of being incredible) idea that one has credible candidate genes, because none of the proposed candidates pass the bar for credibility.

I believe that the palmate-like phenotype should be used a second example of CRISPRs successful implementation, but not as a candidate for nutritional alteration.

8. I wonder if the text on pages 11-13 might be made more concise, without losing the intended meaning?

Reviewer #3 (Remarks to the Author):

Lack of high quality reference genome slows down the fundamental study and breeding of Alfalfa, which is an important forage crop and also is a typical autotetraploidy plant. In this study, the authors de novo assembled a reference of Alfalfa, and also established as CRISPR/Cas9 system. The project is essential, however, current data still need to be improved.

(1) Page 3, line 45, 'Till now, only 5 autopolyploid genomes have been reported', maybe this only refer to plant genomes. Some autopolyploid genomes from animals, particular from fishes have been reported.
(2) The authors clarified that about 81.5% genome were assembled, but 98.8% of the Illumina short reads can map to the genome. Why? Does that mean the Illumina short reads only come from the assembled sequences or the sequencing has some bias? In addition, comparing with current 3rd-

sequencing technology analyses, the Contig N50 is really short. As a good reference, a higher quality is necessary, otherwise, it is hard for other scientists to update it.
(3) The authors annotated more than 80000 protein genes, which is a much larger number. But BUSCO results showed 89% conserved genes can be found. Therefore, the accuracy and completeness of gene annotation also need to be significant improved.
(4) For the chromosome assembly, the authors assembled the autopolyploid genome into two sets of monoploid chromosomes, A and B, which is reasonable and also necessary. There are much more different between A and B, no matter in size, gene number, or TE content. Although the authors made some comparison between them, but still too rough. More detailed comparison needed, for example, why so much difference? Are they coming from deletion or insertion? Which kind of genes were lost from each genome? Which kind of TEs were diverged between them? What is the relationship of each monoploid with M. truncatula genome. What we can get from the divergence? Current data are too basic. Since this is a resource study, a detailed analyses is needed to provide more information for the society.
(5) The second part is establishing CRISPR/Cas9 system. Maybe it is a difficulty for Alfalfa. However, I did not find any closer connection between these two parts. Does the genome promote the establishment of this system? If so, a small paragraph is enough.
(6) Even if the CRISPR/Cas9 system was added, how the system improved should be detailed described and comparison with other system also need to be performed. Otherwise, it is well known that CRISPR/Cas9 system is a quite mature technology which has been popularly used, there is no surprise that it can be used in alfalfa. Instead, the phenotype or transgenic free results do not need such long description, as all these results are expectable.
In summary, sequencing of alfalfa genome is important, but the quality and analyses from current study are not good enough. The second part is not necessary and has not novelty.

Reviewers' comments:

Reviewer #1 (Remarks to the Author):

The manuscript describes results of research to generate a reference genome for the tetraploid obligatory outcrossing plant alfalfa. The author(s) used a combination of single molecule sequencing, genetic maps and Hi-C data to build a reference genome that accounted for roughly 81.5% of the estimated alfalfa genome. They then used the assembled genome sequence to design sgRNA for two genes, PDS and PALM1, for use in CRISPR/Cas9 mutagenesis. With the PDS mutagenesis, they obtained mutants having a dwarf albino phenotype typical of pds null mutants. Further, molecular analysis revealed PDS alleles were indeed mutated in three transgenic plants. In the second CRISPR/Cas9 mutagenesis experiment, they targeted PALM1 gene which is known to control the valuable trait of leaf development (loss of function produces pentafoliate instead of trifoliate), and thus nutritional quality, as proteins accumulate predominantly in the leaves in alfalfa. The authors were able to obtain null palm1 mutants and showed their inheritance in the progeny.

While I do not feel fully qualified to assess the technical aspects of the compilation of the alfalfa reference genome, I recognize assembling this genome is as a major achievement given its large size and tertraploid nature. A number of transcript databases for alfalfa have been published over the years (e.g. Zhang et al, 2015. PLoS One 10:e0122170; O'Rourke et al, 2015, BMC Genomics16:502), but to my knowledge no full alfalfa genome sequence has been made public yet. Researchers have thus had to rely heavily on the genome of the related model plant Medicago truncatula. The publication of the alfalfa reference genome described in this study should therefore allow for major advances in alfalfa research.

Establishing efficient CRISPR/Cas9-based mutagenesis in alfalfa is a major advance, even though previous reports have shown the feasibility of CRISPR/Cas9 mutagenesis in this crop (Gao et al., 2018; Planta 247:1043), albeit with a lower mutagenesis efficiency than reported in this study. The authors followed well established procedures to select sgRNA, make the constructs, and generate/characterize the mutants. What is surprising (and encouraging) is the fact that they obtained null mutants in T0 that were genetically heritable and the pentafoliate phenotype of palm1mutants was stable in the progeny. It is noteworthy that some of the T1 *palm1* mutants were transgene-free. This is sort of unexpected in an organism with a large tertraploid genome and obligatory outcrossing reproductive behaviour.

Reply: We are grateful for these positive comments and suggestions.

Although the manuscript is well written and is technically sound, I have some concerns regarding the following points:

1. Genome editing frequency: It would have been useful to have an idea of the genome editing frequency on a global scale. The authors chose 50 transgenic plants for PDS and 49 for PALM1, for analysis of genome editing using conventional PCR-sequencing technique. A more robust method such as Droplet Digital PCR would have been more informative of genome editing rates/efficiency (Mock et al., 2016, Nat Protoc 11:598).

Reply: We agree with you that we should further clarify this point. High-throughput

mutation-detecting techniques, such as Droplet Digital PCR or targeted deep sequencing, may be more suitable for evaluating frequencies of mutations in cells/protoplasts, since mutations that occur in them cannot be easily detected due to the existence of vast wild-type alleles. However, these technologies are not easily utilized for plant-level analysis, in which the two widely used approaches for evaluating mutagenesis frequencies are calculating ratios of mutants to the total number of transformed calli (Zhang et al., 2016, Nat. Commun. 7:12617; Liang et al., 2017, Nat. Commun. 8:14261), or total number of regenerated plants (Svitashev et al., 2016, Nat. Commun. 7:13274). In the revised manuscript, we have reappraised the genome editing frequency using the total number of calli transformed, as described in the following passages: (p10 lines 211-212): **'***Sequencing chromatograms indicated that five plants were mutants (5/880, 0.57%, designated as mspds1 to 5)***' ; (p11 lines 218-219):'** *while mspds-4 and 5 had mutations in all four alleles (0.23%)***'; (p12 lines 241-243): '***In total, we identified 26 mutants from 1,508 transformed calli (1.72%), including 12 palm1-type plants (0.80%) that developed palmate-like pentafoliate leaves (Fig. 4b and 4c).***'. Thus, the original Fig. 4c has been deleted and the schematic diagram of *MsPALM1* has been moved from Fig. 3 to Fig. 4.**

2. Off-target effects/specificity: The authors assessed off-target effects by screening candidate sgRNA using sgRNACas9 software (i.e. in silico analysis). While this could be considered as a first step towards predicting the presence/absence of off target effects, it does not provide a conclusive proof. To determine off-targets of sgRNA a more comprehensive genome-wide analysis of the mutant by Next Generation Sequencing may be necessary. This is especially true since the authors are relying on a partial assembled genome.
Reply: Thank you for this important suggestion. In the revised version, we resequenced the whole genome of three *palm1*-type mutants (*pa*T0-1, -19 and -46) with 30-fold depth to investigate off-target effects. No off-target mutations were detected in coding regions of these mutants besides the targeted site. This indicates that the specificity of our CRISPR/Cas9-based genome editing protocol can be improved with the help of our genome assembly in designing optimal guide sequences and detecting potential off-targetings. We have added a small paragraph in the main text and Supplementary Table 18 to describe these results (p12 lines 252-260).

3. While the figures are generally well illustrated and presented, I found it hard to follow Figure 3b; it is too crowded with small fonts. Perhaps some of the information can be presented as supplementary data.
Reply: Thanks for pointing this out. In our revised manuscript, we have redrawn Figure 3b to make it clearer and more comprehensible. A high-quality reference genome greatly facilitates application of CRISPR/Cas9 to autotetraploid cultivated alfalfa, by easing cloning, selection of relevant candidate genes and design of optimal guide sequences. Therefore, we think the pipeline (Figure 3b) should be retained in the main figures to clearly display this procedure and connect the genome and genome editing contributions of the study. We have also added corresponding detail about choosing appropriate candidate genes and designing optimal guide sequences in the *Methods* section (p20-21 lines 425-440).

4. The manuscript should undergo at least one more round of editing to correct minor grammar errors. An example would be the word “blasting” on line 443. BLAST is not a verb. The correct wording should be “conducting a BLAST search”.
Reply: We apologize for the grammatical errors. Accordingly, we have proofread the whole manuscript carefully.

Reviewer #2 (Remarks to the Author):
The manuscript entitled, "Chromosome-level reference genome and efficient trangene-free genome editing of autotetraploid alfalfa (Medicago sativa L.0 using CRISPR/Cas9", describes a high quality assembly of a difficulty genetically complex species, deduction of genomic features relevant to the species origin and genome-size expansion, and demonstration of a methodology for CRISPR/Cas9 nucleotide modification.

In general, I am positively inclined towards publication of the document. With the exception of the occasional grammatical error, the document is written in a very approachable style and with appropriate sophistication for a high profile manuscript.

One question that occurred to me several times during my review was, would this manuscript not be better suited by a technology journal such as Nature Biotechnology? I think it is a relevant question, because the authors clearly have genome modification for crop improvement in mind, and despite the apparent high quality nature of the genome assembly this no longer constitutes novel biology which one would normally expect of a Nature Communications article. This comment aside, I provide a set of comments below that the authors might consider in revision.
Reply: Thank you very much for your positive comments and helpful advice. Both *Nature Biotechnology* and *Nature Communications* have very high reputations, and we think our study provides multi-disciplinary contributions that make it more suitable for publication in the multidisciplinary journal *Nature Communications*. In addition, after improving the assembly using recent PacBio CCS technology, we have changed the tittle to “Allele-aware chromosome-level genome assembly and efficient transgene-free genome editing for the autotetraploid cultivated alfalfa”.

1. In the Introduction on page 2 (lines 36-44), the authors are mistaken in the notion that *Medicago truncautla* was developed as a model for alfalfa. This was decidedly NOT one of the rationale. (Perhaps others have subsequently made this statement, but if so then they were mistaken). The sole rationale was to develop a tractable legume to study symbiosis. To the extent that *Medicago truncatula* was envisioned as a model for legumes, this came later and even then we thought of truncatula being a model for all legumes (not alfalfa in particular). It was only with the involvement of the Noble Foundation, and a few colleagues at USDA-ARS, that the idea of using truncatula as a model for sativa gained traction (and then it remained a small part of a larger rationale).
OK, sorry for the history lesson, but please revise the second paragraph in the discussion to remove statements about the rational for *Medicago truncatula*.
Reply: Thank you very much for this professional advice. We have rewritten this sentence to improve its accuracy, as follows (p2 lines 36-38): ‘*Previous exploration of genetic and genomic*

resources of alfalfa mostly relies on its close relative, the diploid M. truncatula (2n=2x=16=860 Mb) which has been sequenced.'

2. On page 4 (lines 72-72), the authors state that 89.1% of BUSCO homologs were identified. But it seems important to distinguish cases were all orthologs (4X) were encountered (which would be the actual rate of discovery), versus cases where assembly was adequate to capture only a subset (corrected for rates of actual loss).
Reply: Good point. In the revised version, we significantly improved the genome assembly, and found 97.2% of single-copy orthologous genes in a monoploid genome were retrieved by BUSCO analysis. It is currently the most complete autotetraploid genome assembly, in which 90.11% of BUSCO genes are duplicated and 7.05% are single-copy, compared with 81% and 14.4%, respectively in the published polyploid sugarcane genome. These results indicate that the assembled genome has relatively high accuracy and completeness. We have added a corresponding description and Supplementary Table 9 in the revised manuscript (p5 lines 100-103) to present this analysis more clearly.

3. Page 5, lines 86-87. Note that the chromosome 4 vs 8 translocation that the authors report is a genomic feature specifically of the Medicago genotype (A17) that was sequenced in the truncatula genome project. The base state of the truncatula species does not have this rearrangement. I think it is important to clarify this.
Reply: Thanks for pointing this out. Yes, the translocation has been reported to occur in the *Medicago truncatula* A17 genome. Based on your suggestion, in the revision, we have deleted the statement and the previous Fig 2a about this issue to avoid the misleading that this is a novel discovery in our study.

4. Page 8, line 116, and Figure 2d. It is not possible from Figure 2d for the reader to understand that certain classes of reverse transcriptases are expanded in the Ms subgenomes relative to Mt. I would suggest two corrections: 1. Improve the clarity of the figure, and 2. Provide a table in which the RTs are divided into clades and in each clade the frequency of Msa, Msb vs Mt homologs are enumerated – or some other quantitative presentation to back up the authors assertion about TE expansion.

Also, in the same paragraph, line 123, please provide the quantitative data that TE insertions are the main reason for genome expansion. What is the size difference of each subgenome relative to Mt and what percent of the size difference is attributable to TEs?
Reply: Using the newest genome assembly, we re-analyzed the TE contents in each subgenome of *M. sativa* and *M. truncatula*, and accordingly updated Supplementary Table 13 and removed Fig. 2d in the revised manuscript, which shows that allelic chromosomes have similar TE contents. We also summarized differences in TE contents between *M. truncatula* and *M. sativa* in Supplementary Table 14, which indicates that differences in sizes of intergenic regions contributed most to the expanded *M. sativa* genome, and Ty3/Gypsy, non-repeat, Ty1/Copia, simple/tandem repeat and DNA elements explain 31.93, 26.57, 13.56, 9.93 and 6.87% of the total size difference, respectively, collectively explaining 88.85% of the total difference in genome size between Msa and Mt (p9 lines 177-182).

5. Page 8, lines 124 and on. I am a bit concerned about the phylogenetic analyses. Given that all legumes under comparison derive from an ancient polyploidization event, followed by diploidization, then the usefulness of singleton orthologs for phylogenetic analysis will be a function of the timing of loss relative to speciation (i.e., whether or not the retained singletons represent a coherent common history or not). I know from previous work that in some cases, return to a single copy state occurred after speciation, with different paralog histories in the different genomes.

Assuming that I am expressing my concern adequately, then my question, how can one derive a realistic phylogenetic analysis? I am struck by the accuracy of the resulting tree, which suggests to me that these genes became single copy before the species radiated. Is there evidence of this in the data? There are methods for looking at phylogenetic coherence between genes and I think that this is an important issue.

Reply: Thank you for this important suggestion. Orthologs are the most widely used molecular markers for inferring phylogenetic relationships, as they record the real divergence history between considered species. While acknowledging that complex polyploidization events occurred, we totally agree that the identification of the single-copy genes may be difficult in polyploid species. To avoid this problem in such a tetraploidy, we applied another two methods in the revised version. One was the BUSCO pipeline for phylogenetic analysis (Waterhouse et al., 2018, Molecular Biology and Evolution, 35(3): 543-548), selecting the most completely sequenced genes for each highly conserved BUSCO gene set in each species. The other was to extract low-copy genes (less than 10 copies in each species) to construct gene family trees, followed by use of STAG (Emms et al., 2019, Genome Biol., 238) to construct a species tree. Both sets of results show identical topology, which may reflect the stable relationships in legumes.

6. Page 7, line 132, the basal nature of Arachis and Lupinus is not novel, nor in question, and should not be presented as if this is a novel result. What you have learned is that your analysis recapitulates known relationships.

Reply: We agree that the basal nature of *Arachis* and *Lupinus* is not novel, and we have extensively rewritten the related sentences in the revision. In our new phylogeny analysis, the results provide bootstrap values and shows that posterior probabilities of the basal lineages are low while the other node have very high support. We have added phylogenetic results from another two datasets (low-copy genes and conserved BUSCOs) to elucidate the most realistic relationships among legume species. We found a very short time of divergence (~4.58 Mya) of the ancestral legume into *Arachis* and *Lupinus*, and the low robustness of the topology may be due to associated radiative diversification. In the revised manuscript we have included three new figures (Supplementary Figures 8-10) to more clearly present this issue.

7. Page 8 lines 150-151 and line 155, page 10 lines 207-210, and page 13 lines 258-259. The notion that specific gene functions might be well enough understood or impactful enough to modulate nitrogen fixation, abiotic stress or nutrition is overly speculative. Certainly these can be

described as traits that one might be interested in modifying, but there is no credible evidence that DNA repair (for example) might improve nutritional quality or that simply altering leaf morphology will have a significant impact on nutritional value. (I am aware of the Palmate mutants in Mt and I consider the role in plant nutrient content to be significantly oversold). Similarly, the suggestion that finding 2 copies of 22 sym genes (in Ms relative to Mt) might lead to insights in nitrogen fixation is very, very over simplistic.
Reply: We are grateful for these valuable comments and agree that analyses of specific genes' functions and GO functional enrichment are not sufficient for definitive conclusions regarding phenotypic effects of modifications. We have therefore removed these statements and their subsequent discussions to avoid the problem overstatement.

If feel quite strongly that one needs to separate the proof that one can modify genetic targets with CRISPR, which the authors show with data, from the highly speculative (to the point of being incredible) idea that one has credible candidate genes, because none of the proposed candidates pass the bar for credibility.
I believe that the palmate-like phenotype should be used a second example of CRISPRs successful implementation, but not as a candidate for nutritional alteration.
Reply: Thank you for these important suggestions to improve our manuscript. We agree that we should tone down the suggestion that simply altering leaf morphology will significantly affect nutritional values. As null *palm1* mutants of *M. truncatula* developed palmate-like pentafoliate leaves with higher than wild type leaflet numbers, now we just tentatively hypothesize that editing *MsPALM1* of cultivated alfalfa may increase the leaflet number of the leaves and provide an option to breed multileaflet alfalfa varieties, although further studies are required to test whether the increased leaflet number of *palm1*-type mutants results in improved leaf biomass and higher forage quality. And as you pointed out, *MsPALM1* provides another ideal candidate gene to test the stability of our CRISPR/Cas9-based genome editing protocol since morphological changes in leaf are easily visible phenotypic markers.
In our revised manuscript, we have rephrased and toned down the related description as follows: (p11, lines 226-236): *'A high leaf/stem ratio is an important agronomic trait for cultivated alfalfa, as it is positively correlated to the nutritional value of alfalfa products. Breeding varieties with more leaflets per leaf may improve the leaf/stem ratio of cultivated alfalfa and thus increase its yield and nutritional value. In diploid M. truncatula, PALM1 encodes a Cys(2)His(2) zinc finger transcription factor that plays a key role in compound leaf morphogenesis. Null palm1 mutants develop palmate-like pentafoliate leaves rather than wild-type trifoliate leaves. Thus, we hypothesized that disruption of PALM1 orthologs (MsPALM1) in cultivated alfalfa may enable it to express the palm1 phenotype. This would also provide another easily visible example to validate the stability of our protocol and its potential for generating multileaflet varieties.'* **and (p13 lines 275-280)**: *'In addition, the generation of these transgene-free palm1-type progenies indicates that CRISPR/Cas9 technology may provide a shortcut for breeding multileaflet varieties which may have higher nutritional value, although further studies are required to test whether the increase in leaflet number is accompanied by improvements in leaf biomass and forage quality.'*

8. I wonder if the text on pages 11-13 might be made more concise, without losing the intended

meaning?

Reply: We have compressed this passage by discarding redundant information and descriptions of technology, only retained the necessary results of experiments and their implications (p11-13, lines 237-280).

Reviewer #3 (Remarks to the Author):

Lack of high quality reference genome slows down the fundamental study and breeding of Alfalfa, which is an important forage crop and also is a typical autotetraploidy plant. In this study, the authors de novo assembled a reference of Alfalfa, and also established as CRISPR/Cas9 system. The project is essential, however, current data still need to be improved.

Reply: We are grateful for the reviewer's positive comments.

(1) Page 3, line 45, 'Till now, only 5 autopolyploid genomes have been reported', maybe this only refer to plant genomes. Some autopolyploid genomes from animals, particular from fishes have been reported.

Reply: Thank you very much for pointing out this issue. This sentence has been accordingly rephrased as follows (p2 lines 41-42): *'So far, only five plant autopolyploid genomes have been reported.'*

(2) The authors clarified that about 81.5% genome were assembled, but 98.8% of the Illumina short reads can map to the genome. Why? Does that mean the Illumina short reads only come from the assembled sequences or the sequencing has some bias? In addition, comparing with current 3rd-sequencing technology analyses, the Contig N50 is really short. As a good reference, a higher quality is necessary, otherwise, it is hard for other scientists to update it.

Reply: Thank you for this important suggestion. The main reasons for our previous assembly having a low genome size but high read mapping rate are the bias of Illumina sequencing (as you pointed out) and the collapse of repetitive sequences in the assembly, which is quite common in genome assembly and could down-size the assembly without influencing the mapping rate. We admit that the contig N50 is short, although it was longer than N50 values obtained for other released autopolyploid plant genomes. Based on your suggestion, in the revision we have generated new data using a recent high fidelity single molecule sequencing technology (PacBio CCS) and reassembled the cultivated alfalfa genome, and significantly improved the assembly in all aspects. The contig N50 is 459 kb, longer than our previously assembly and published assemblies for sugarcane (45 kb) and sweet-potato (3.5 kb) genomes. More importantly, we successfully assembled these contigs into four sets of allele-aware chromosomes using Hi-C data, given that the autotetraploid cultivated alfalfa has a tetrasomic inheritance in which bivalent pairing is random and not preferential (Dilkova an Bingham, 2017). The allele-aware chromosome-level scaffolds are of high quality, as revealed by ONT (Oxford Nanopore Technology) long reads check, Hi-C heatmapping, genetic linkage mapping and synteny block alignment, and thus will be very useful in the future study and breeding on the cultivated alfalfa.

(3) The authors annotated more than 80000 protein genes, which is a much larger number. But BUSCO results showed 89% conserved genes can be found. Therefore, the accuracy and

completeness of gene annotation also need to be significant improved.

Reply: This is a very good question and made us analyze the results in a more comprehensive way. In our newly assembled genome, all four allelic chromosomes are assembled. Now we obtained a total of 164,632 genes from this assembled tetraploid genome, in which more than 97% complete BUSCOs could be retrieved. In other words, the annotated gene number were from all genes in the whole tetraploid genome, unlike other reported monoploid genomes, which usually contains about 4,000 genes. We found similar gene characters (such as gene length, intron length, exon number) between alfalfa and *Medicago truncatula*, and more than 95% predicted genes have homologs in NR, GO, KEGG, InterProscan, Swissprot and TrEMBL databases.

(4) For the chromosome assembly, the authors assembled the autopolyploid genome into two sets of monoploid chromosomes, A and B, which is reasonable and also necessary. There are much more different between A and B, no matter in size, gene number, or TE content. Although the authors made some comparison between them, but still too rough. More detailed comparison needed, for example, why so much difference? Are they coming from deletion or insertion? Which kind of genes were lost from each genome? Which kind of TEs were diverged between them? What is the relationship of each monoploid with M. truncatula genome. What we can get from the divergence? Current data are too basic. Since this is a resource study, a detailed analyses is needed to provide more information for the society.

Reply: Thank you very much for your comments and suggestions, which have prompted us to check more literature and realize the autotetraploid cultivated alfalfa actually has a tetrasomic inheritance in which bivalent pairing is random and not preferential (Dilkova an Bingham, 2017). This means that we cannot artificially separate the assembly into A and B groups, and rather we should assemble all the four homologous chromosomes for the variety we used. So, we decided to use the PacBio circular consensus sequencing (CCS) technology to get single molecular long reads with high fidelity which has comparable error rates to NGS, and thus allowed us to extend contigs from highly similar sequences. Using the ALLHiC algorithm which is capable of building allele-aware, chromosome-scale assembly for autopolyploid genomes together with Hi-C paired-end reads (Zhang et al., 2019, Nat Plants, 5, 833-845), we indeed successfully assembled the allele-aware chromosome-level genome for the autotetraploid cultivated alfalfa. We evaluated the assembly by ONT (Oxford Nanopore Technology) long reads check, Hi-C heatmapping, genetic linkage mapping, BUSCO analysis and synteny block alignment, and found the assembly is of quite high quality. We observed that the genome size, gene number and TE contents are similar within each monoploid chromosome group. The heterozygosity between any two monoploid genome sets was estimated with an average value of 1%, similar to that of diploid alfalfa. We also analyzed overall gene expression differences among 4 homologous chromosome groups by sampling genes with four alleles and found no significant difference among homologous chromosomes. These results are consistent with the fact that this species has a tetrasomic inheritance in which bivalent pairing is random and non-preferential. In our revised manuscript we have rephrased the relevant sections accordingly (p6 line 110-p7 line 138), and added one figure (Supplementary Figure 7) and one table (Supplementary Table 13) to display these new results.

(5) The second part is establishing CRISPR/Cas9 system. Maybe it is a difficulty for Alfalfa. However, I did not find any closer connection between these two parts. Does the genome promote the establishment of this system? If so, a small paragraph is enough.

Reply: We are sorry for not clearly describing the connection between the two parts. Yes, decoding the genome can greatly facilitate application of genome editing technologies. Without a reference genome, it is very difficult to clone and choose appropriate candidate genes, and extensive efforts are needed to clone such genes by traditional genetic approaches or PCR analyses based on limited information. These are time-consuming processes, especially for members of gene families or redundant genes. With the help of a high-quality reference genome, we can shorten the time required to obtain comprehensive information about candidate genes, choose appropriate candidate genes and design optimal guide sequences that have the fewest potential off-target sites and are located in the regions near their start codons. In addition, we can globally evaluate off-targeting by Next Generation Sequencing with the availability of genome assembly. In our revised manuscript, we have highlighted the pivotal role of the genome in promoting the application of CRISPR/Cas9-based genome editing and connect the complementary genome and genome editing parts of the study, as follows (p 9 lines 189-194): '*The allele-aware chromosome-level cultivated alfalfa genome assembly obtained in this study provides a necessary start point to accurately apply the CRISPR/Cas9 technology to help in screening candidate genes, decoding gene structural information and designing optimal guide sequences (Fig. 3b, detailed in methods). Conversely, this genome editing technology could help efforts to convert the enormous amount of genome data into functionally relevant knowledge.*' **In addition, we have redrawn Figure 3b to illustrate how to clone candidate genes and design optimal guide sequences with the help of the high-quality reference genome.**

(6) Even if the CRISPR/Cas9 system was added, how the system improved should be detailed described and comparison with other system also need to be performed. Otherwise, it is well known that CRISPR/Cas9 system is a quite mature technology which has been popularly used, there is no surprise that it can be used in alfalfa. Instead, the phenotype or transgenic free results do not need such long description, as all these results are expectable.

In summary, sequencing of alfalfa genome is important, but the quality and analyses from current study are not good enough. The second part is not necessary and has not novelty.

Reply: Thank for your comments. Yes, we agree CRISPR/Cas9 is a quite mature technology. In the case of the cultivated alfalfa, breeding and study are still of difficulty due to the lack of whole genome information and effective gene-editing pipeline. The feature of autotetraploidy and self-incompatibility have imposed some inherent challenges to efficiently and accurately edit 4 copies at the same time. Any mutant generated by gene-editing has not been reported for the cultivated alfalfa, therefore it would be beneficial to simultaneously provide a good genome assembly and an initial gene-editing protocol for the cultivated alfalfa community, so that other researchers can start with it and save time and resources in try and error process. And as you suggested, we have toned down this part and shortened the relevant sentences about phenotypes and transgenic sequence-free results in our revised

manuscript.

References

1. Zhang, Y. et al. Efficient and transgene-free genome editing in wheat through transient expression of CRISPR/Cas9 DNA or RNA. *Nat.Commun.* **7**, 12617 (2016).
2. Liang, Z. et al. Efficient DNA-free genome editing of bread wheat using CRISPR/Cas9 ribonucleoprotein complexes. *Nat. Commun.* **8**, 14261 (2017).
3. Svitashev, S., Schwartz, C., Lenderts, B., Young, J.K. & Mark Cigan, A. Genome editing in maize directed by CRISPR-Cas9 ribonucleoprotein complexes. *Nat. Commun.* **7**, 13274 (2016).
4. Waterhouse, R.M. et al. BUSCO Applications from Quality Assessments to Gene Prediction and Phylogenomics. *Mol. Biol. Evol.* **35**, 543-548 (2018).
5. Emms, D.M. & Kelly, S. OrthoFinder: phylogenetic orthology inference for comparative genomics. *Genome Biol.* **20**, 238 (2019).
6. Dilkova, M. & Bingham, E. Microsporogenesis of Alfalfa Cultivars and Selected Genotypes II. *Medicago Genetics Reports* www.medicago-reports.org **17**, 1-16 (2017).
7. Zhang, X., Zhang, S., Zhao, Q., Ming, R. & Tang, H. Assembly of allele-aware, chromosomal-scale autopolyploid genomes based on Hi-C data. *Nat. Plants* **5**, 833-845 (2019).

REVIEWERS' COMMENTS:

Reviewer #1 (Remarks to the Author):

I thank the authors for addressing my comments/concerns.

Reviewer #2 (Remarks to the Author):

The authors provided a substantive response to each of my initial comments and I am satisfied with the ways in which they have addressed my comments. On that basis, I am in favor of the manuscript being accepted for publication.

Reviewer #3 (Remarks to the Author):

The revised manuscirpt has answer all my questions. I do not have any more comments.

Reviewer #1 (Remarks to the Author):
I thank the authors for addressing my comments/concerns.
Response: We are grateful for your comments during our revising procedures.

Reviewer #2 (Remarks to the Author):
The authors provided a substantive response to each of my initial comments and I am satisfied with the ways in which they have addressed my comments. On that basis, I am in favor of the manuscript being accepted for publication.
Response: We are grateful for your comments during our revising procedures.

Reviewer #3 (Remarks to the Author):
The revised manuscript has answer all my questions. I do not have any more comments.
Response: We are grateful for your comments during our revising procedures.
